# Molecular Simulation Strategies for Understanding the Degradation Mechanisms of Acrylic Polymers

**DOI:** 10.1021/acs.macromol.2c02442

**Published:** 2023-04-19

**Authors:** Aysenur Iscen, Nancy C. Forero-Martinez, Omar Valsson, Kurt Kremer

**Affiliations:** † Max Planck Institute for Polymer Research, Ackermannweg 10, 55128 Mainz, Germany; ‡ Department of Chemistry, University of North Texas, Denton, Texas 76203, United States

## Abstract

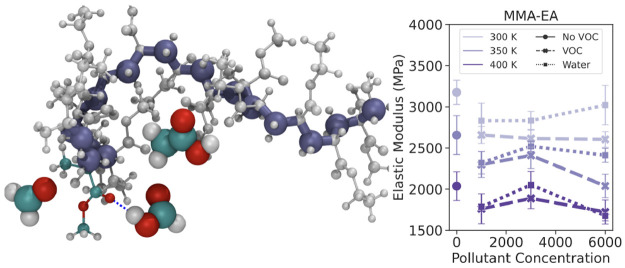

Acrylic polymers, commonly used in paints, can degrade over time by several different chemical and physical mechanisms, depending on structure and exposure conditions. While exposure to UV light and temperature results in irreversible chemical damage, acrylic paint surfaces in museums can also accumulate pollutants, such as volatile organic compounds (VOCs) and moisture, that affect their material properties and stability. In this work, we studied the effects of different degradation mechanisms and agents on properties of acrylic polymers found in artists’ acrylic paints for the first time using atomistic molecular dynamics simulations. Through the use of enhanced sampling methods, we investigated how pollutants are absorbed into thin acrylic polymer films from the environment around the glass transition temperature. Our simulations suggest that the absorption of VOCs is favorable (−4 to −7 kJ/mol depending on VOCs), and the pollutants can easily diffuse and be emitted back into the environment slightly above glass transition temperature when the polymer is soft. However, typical environmental fluctuations in temperature (<16 °C) can lead for these acrylic polymers to transition to glassy state, in which case the trapped pollutants act as plasticizers and cause a loss of mechanical stability in the material. This type of degradation results in disruption of polymer morphology, which we investigate through calculation of structural and mechanical properties. In addition, we also investigate the effects of chemical damage, such as backbone bond scission and side-chain cross-linking reactions on polymer properties.

## Introduction

Conservation of contemporary artworks presents a challenge because of the variety and complexity of materials used by modern artists. The basic rule for conservation (preservation and stability) of cultural heritage works is to know the artwork’s composition and the decay processes that can occur and lead to the degradation of the art object to implement a conservation treatment.^1,2^

Elucidation of the materials’ composition requires different analytical techniques to classify them generally or obtain a more detailed chemical characterization. In the case of synthetic composites, and more explicitly acrylic paints frequently used in contemporary art, techniques such as Fourier transform infrared spectroscopy,^3,4^ pyrolysis–gas chromatography, and mass spectrometry^4−6^ are widely used to obtain information about major components such as binders^4,7^ and pigments.^8^ However, these techniques are not enough to identify intrinsic materials characteristics and how they respond to environment to guide conservators and scientists to design more suitable techniques and/or treatments for the preventive conservation of art objects.

Only recently, the effects of aging in artworks based on synthetic materials are becoming apparent,^4^ and there is a clear need to understand the degradation mechanisms that lead to visual changes on artworks based on synthetic materials to support their preventive conservation. Preventive conservation of cultural heritage objects is the focus of recent studies resulting in the development of new materials to provide art objects with a protective layer against deterioration sources,^9^ such as ultraviolet (UV) light,^10^ oxygen, moisture, and corrosive agents. Moreover, these studies allow for the monitoring of volatile organic compounds (VOCs)^11^ connected to corrosion, cross-linking, or discoloration^12−14^ of exposed artworks in museums and allow for the design of smart storage of museum artifacts, providing microclimate monitoring.^15^ In this context, it makes sense to focus on synthetic materials, specifically acrylic paints, because their stability and response to aging depend on various factors. While some properties of acrylics are inherent to the material, such as composition, glass transition, modulus of elasticity, and viscosity of the paint film,^16^ others are due to handling and processing, such as manufacturing technology, ambient conditions while drying, and exposure to air pollutants and humidity.^1^ Therefore, degradation of acrylic paints is a complex phenomenon that originates from different sources. In this context, we devote this study to investigating different degradation mechanisms in the acrylic polymer binder.

Acrylic paint is a mixture of pigment particles dispersed in an emulsion created by water and small amorphous polymer beads. Acrylic paints dry through a process called polymer coalescence. Once applied, e.g. onto the canvas, the water evaporates or is absorbed into the surface. As the paint dries, the polymer particles are drawn closer together, creating a stable film that traps the pigment.^16,17^ Once dry, acrylic polymer regions with sizes between 50 and 500 nm^18^ separate the pigment particles. Since the first commercially available waterborne acrylic paint, Liquitex,^2^ artists continue to use them due to their desirable characteristics: paint film clarity, easy manipulation and application, fast drying times, dilution with water, versatility, flexibility, and high resistance to ultraviolet damage.^2,19^ Early acrylic emulsion formulations such as those based on copolymers made of methyl methacrylate (MMA) and either ethyl acrylate (EA) monomers (RhoplexAC-33^18^) or *n*-butyl acrylate (nBA) monomers were designed to exhibit glass transition (*T*_g_) temperatures below or near room temperature to avoid cracking at lower than ambient temperatures.^20,21^ Today, paint manufacturers continue testing and improving professional quality paints to address issues such as lack of rewetting ability and wet-to-dry color shift. New formulations include the replacement of the nBA monomer with 2-hydroxyethyl acrylate (2-HEA) and addition of 2-HEA to (PMMA-*co*-nBA).^22,23^

In the context of polymer science, we understand degradation as any change in the polymer structure which affects the mechanical, chemical, optical, or electrical properties of the polymer material.^24,25^ These changes are either of chemical nature, due to breaking and forming chemical bonds, or changes in the physical rearrangement of the polymer chains. In the case of acrylic polymers, degradation agents include heat, exposure to UV light,^26^ interaction with atmospheric gases and air pollutants,^12−14^ hydrolysis, and, less likely, biodegradation.^27^ The polymer response to these agents depends strongly on the polymer state, glass transition temperature, presence of ester groups (−CO–O−), and tertiary hydrogen atoms. Ester groups make the polymer susceptible to hydrolysis, while CH groups make the acrylics sensitive to reactions when free radical species are present.^25^

Degradation reactions in polymers fall into two broad categories: those occurring due to thermal stress (heat application) or as a response to ionizing radiation (e.g., ultraviolet light). In both cases, reactions might involve bond homolysis either in the backbone or on the side groups attached to the backbone. Thermal degradation leads to depolymerization and scissions or cyclization of the side groups.^25^ However, degradation of acrylic polymers by depolymerization is not significant at ambient temperatures.^28^ In the case of photodegradation, the UV light has enough energy to break covalent bonds with energies between 300 and 500 kJ/mol (e.g., C–C and C–O bonds). Photodegradation pathways result from light absorption by a chromophore within the polymer or by an additive in the paint. Hence, excited polymer molecules undergo homolysis, namely, backbone or group side scission and cross-linking. Predicting patterns of thermal and photodegradation is still a difficult task. However, some studies relate the polymer structure with the aging mechanism, suggesting that chain scission reactions are primarily responsible for degradation in P(MMA-*co*-EA) while cross-linking is responsible in P(MMA-*co*-nBA).^29,30^

Changes in the mechanical properties account for the effects of degradation in acrylic polymers. Quantities such as yield stress, tensile strength, and Young’s modulus, in combination with spectroscopic techniques and molecular weight measurements, describe the structural and molecular changes occurring under degradation.^4^ Strain versus stress measurements resolve these changes. Chiantore et al.^31^ observe that the elastic modulus increases with exposure time for P(MMA-*co*-EA) subjected to artificial sunlight. At the same time, tensile strength decreases, pointing to a weak and brittle film after photoaging. Analytical characterization of different colors and brands of acrylic paints shows that mechanical properties are sensitive to paint composition, particularly pigment content.^32,33^ While Young’s modulus of samples lacking pigment does not seem affected by aging, colored films show a decreased stiffness and, therefore, lower elastic constant for samples at different aging times. Other factors affecting mechanical properties are temperature and water content.^33^

In our previous study,^34^ we developed a computational model to focus on how volatile organic compounds (VOCs) and water in the environment interact with the acrylic polymers found in modern paints. Our model was an atomistic representation of two types of acrylic binders, P(MMA-*co*-EA) and P(MMA-*co*-nBA), which make up the majority of the acrylic paints. We analyzed temperature-dependent structural properties and calculated glass transition temperatures. We also investigated the interaction of acetic acid, formic acid, formaldehyde and water with the acrylics. In this paper, we explore the absorption/desorption dynamics of VOCs into the acrylics from the environment in more detail by calculating the free energy of absorption around glass transition temperature using enhanced sampling methods. This gives us first quantitative information about how likely it is for VOCs to accumulate on the surface of acrylics and/or be absorbed over time. In addition, we also discuss how different degradation mechanisms, such as absorption of pollutants, backbone bond scission, and side-chain cross-linking reactions, give rise to changes in mechanical properties of these materials. We believe that the microscopic insight we gain from these simulations will not only be of interest in the cultural heritage conservation community but also apply to a broader audience because acrylic polymers are commonly used in many products including paints, coatings, textiles, adhesives, and plastics.

## Methods

This work is a continuation of our previous work, where we developed an atomistic model for acrylic polymers found in the paints. We study two separate systems: copolymer chains made up of 40% MMA and 60% EA (or nBA). This ratio of polymers in the copolymer was based on experimentally determined compositions of binders used in acrylic paints: Rhoplex (Primal) AC-33 and AC-235 by Rohm & Haas.^7,16,35^ Each copolymer chain has 15 monomers, and each system contains 100 polymer chains. In order to introduce some randomness into our copolymers, we modeled five different copolymer chains with the same composition by only changing the order of the monomer sequences. The initial box size was 9 × 9 × 9 nm^3^. Simulations were performed using the General Amber force field (GAFF)^36^ with the Gromacs 2019.4^37,38^ software. For more information on the models and simulation details, please refer to Iscen et al.^34^ A summary of all the simulations presented in this study is included in Table S1.

### Freestanding Films

In addition to the bulk simulations performed previously, we constructed copolymer films using the equilibrated bulk simulation at 600 K and extending the box size in the *z*-dimension to 20 nm. This allowed us to eliminate contact of the polymer chains along the *z*-axis and form two stable surfaces exposed to a vacuum. After short equilibration at 600 K (10 ns), the same cooling procedure as before (with cooling rate of 1.2 × 10^12^ K/min or 20 K/ns) was applied with the exception of using the NVT ensemble. This allows the formation of copolymer films with different thickness, which results in a change in density during the cooling step (Figure S1). Following the cooling step, the copolymer films were equilibrated for 100 ns at each temperature (100–600 K with 50 K intervals) using the NVT ensemble.

In order to study the absorption of pollutants from the environment into the films, 10 molecules of each pollutant (acetic acid, formic acid, formaldehyde, and water) were placed in the vacuum between the copolymer film surfaces. For initial structures, we used the copolymer film coordinates after 100 ns of equilibration at each temperature (250–500 K with 50 K intervals). This was done in order to guarantee the structure of the copolymer films was consistent with previous results and did not depend on the presence of VOCs outside of the film. The films with pollutants were equilibrated for 10 ns at each temperature.

In order to determine whether the film thickness has any effect on the absorption and diffusion of the pollutants, we also prepared thin films using twice the number of polymer chains (200 chains). These films were prepared using the same procedure, where we first equilibrated 200 polymer chains in bulk using the NPT ensemble for 100 ns. After confirming that the glass transition temperature is similar for both bulk and thin film polymer simulations (Figure S2), we prepared the films by extending the simulation box in the *z* dimension of the equilibrated polymer at 600 K. We cooled the films to 100 K using the previously specified cooling rate and further equilibrated the films for another 100 ns using the NVT ensemble. After the films were equilibrated, we inserted pollutants in the vacuum space between the film surfaces and performed additional 10 ns of unbiased MD simulations in order to investigate the absorption and diffusion of VOCs into thin films. As a result of these simulations, we did not observe a significant effect of system size on the film properties or pollutant diffusion (Figure S3). Furthermore, we also tested the effect of cooling rate on thin film properties by performing separate simulations for thin films with 100 polymer chains using cooling rates of 10 and 5 K/ns in addition to 20 K/ns. The change in the *T*_g_ was within error because we have simulations at 50 K intervals, i.e., at 400 and 450 K (Figure S4).

For all simulations, bonds involving hydrogen atoms were constrained using the LINear Constraint Solver (LINCS) algorithm.^39^ The Verlet cutoff scheme^40^ was used for neighbor searching. Long-range electrostatics were determined with the smooth particle mesh Ewald (PME)^41^ method using cubic interpolation and Fourier grid spacing of 0.16 nm. A cutoff of 1 nm was used for evaluation of all nonbonded interactions. Atomic coordinates were saved every 100 ps for the trajectory analysis. Each system was minimized for 1000 steps using the steepest descent algorithm. For the NVT ensemble, we used the v-rescale coupling method^42^ and a 2 fs time step.

### Metadynamics Simulations

The free energy surface for VOC absorption/desorption into acrylic films was calculated with the well-tempered metadynamics method^43,44^ using GROMACS 2019.6^37,38^ molecular dynamics (MD) code and the PLUMED 2.5.4^45,46^ enhanced sampling plug-in. For this, we used freestanding films of P(MMA-*co*-EA) previously equilibrated at different temperatures for 100 ns. The size of the film was 6.5 nm × 6.5 nm and continuous with periodic boundary conditions in *x* and *y* directions. The films were ∼4 nm in thickness, and the size of the simulation box was 20 nm along the *z* direction to allow for vacuum on both sides of the film. For each simulation one VOC (acetic acid, formic acid, or formaldehyde) was placed in vacuum at a random distance away from the film. We performed the metadynamics simulations using the multiple-walker method^47^ with eight walkers running in parallel, where the initial distance between VOCs and film was different for each walker. We used the *z* component of distance between the center of mass of the film (carbon atoms) and the center of mass of the VOC (carbon atoms) as our collective variable. During the simulations, the bias factor was 160, the Gaussian deposition pace every 500 steps, height 2.5 kJ/mol, and width 0.4 nm. We ran two independent simulations 100 ns long for each walker (a total of 1.6 μs for each VOC) at 300 K (below *T*_g_) and 450 K (above *T*_g_). For each simulation, the free energy surface was obtained using the *c*(*t*) reweighting procedure^44,48^ where we disregarded the first 5 ns of each walker. Because we do not want to distinguish between absorption/desorption on the two sides of the film, we assumed the film density to be homogeneous throughout and calculated the free energy surface as a function of absolute value of distance from the film center.

The time evolution of the collective variable and Gaussian hill height for the metadynamics simulations at 300 and 450 K are shown in Figures S5–S8. These figures show that the transitions of VOCs in/out of the polymer film are rare at 300 K due to glassy state of the polymers, but frequent at 450 K, which is slightly above the simulated glass transition temperature for P(MMA-*co*-EA). In all simulations, we can visualize the decrease of the Gaussian height during the simulation from an initial value of 2.5 to 0 kJ/mol. We also calculated the error from each simulation using the block averaging method. In all cases, the maximum average error was around 0.2–0.3 kJ/mol (for example, see Figure S9).

### Deformation Simulations

In order to calculate stress versus strain curves, we performed nonequilibrium MD simulations for our bulk copolymer systems already equilibrated for 100 ns. We deformed our equilibrated boxes at each temperature by applying a uniaxial strain, which changes the periodic cell size at a constant rate along one of the axes (*x*, *y*, or *z*). We performed the deformation along each axis separately using straining rates of 0.1, 0.01, and 0.001 nm/ps. During these simulations, the isotropic pressure coupling was replaced with anisotropic Berendsen coupling with τ_p_ = 1 ps, and the reference pressure and compressibility values were set to 0.0 for the axis along which the simulation box was deformed. During the deformation, the values of the pressure tensor (*P*_*i*,*i*_ where *i* = *x*, *y*, or *z*) and the simulation box size *L*_*i*_ were saved every 1 ps. Each deformation simulation was 8 ns long, which was long enough to observe at least 100% strain for all strain rates. Stress (σ) and strain (ϵ) were calculated as follows from simulations where deformation was performed in *x*, *y*, and *z* directions:
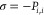
1
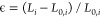
2where *L*_0,*i*_ is the initial size of the simulation box in the deformation direction *i* = *x*, *y*, or *z*. Stress and strain values from three separate simulations (*x*, *y*, and *z* deformation) were averaged for all of the strain versus strain curves presented in this paper. Because of large fluctuations in the pressure tensor at low strain rates, the average values of stress and strain were plotted along with running averages calculated from every 200 ps of data. The initial part of the stress versus strain plots shows a linear dependence up to about 4% deformation, and the elastic modulus (*E*) was calculated from the slope of the line σ = *E*ϵ up to ϵ = 4%. The error bars were calculated from the standard error of mean of calculated *E* in *x*, *y*, and *z* deformation directions.

We also performed deformation simulations of the bulk copolymers with additional pollutants. For this, we used previously equilibrated simulations^34^ with 1000, 3000, and 6000 ppm VOCs (acetic acid, formic acid, and formaldehyde) or water at 300, 350, and 400 K to demonstrate the effect of the pollutants on mechanical properties around the glass transition temperature.

### Bond Scission Simulations

One of the mechanisms of damage in acrylics is bond scission reactions. Because classical molecular dynamics simulations with nonreactive force fields lack the ability to simulate the chemical reaction that happens during damage, we took an alternative approach that would simplify the bond scission reaction. Using our bulk simulations of copolymers equilibrated for 100 ns at 600 K, we made cuts between 10th and 11th monomers in the polymer backbone to turn all of the 15-mer copolymer chains into 10-mer and 5-mer chains (Figure 5). However, we did not account for the missing hydrogens at the chain ends where the cuts were made. After making this change in the topology, we did a short minimization (1000 steps), NVT equilibration (200 ps), and NPT equilibration (10 ns) at 600 K, allowing the polymer chains to relax. This equilibration was followed by the cooling procedure (with cooling rate of 1.2 × 10^12^ K/min or 20 K/ns) from 600 to 100 K. The coordinates of the polymers were saved for each temperature in the temperature range 250–500 K with 50 K intervals. We equilibrated each temperature for an additional 20 ns in the NPT ensemble before deformation simulations using straining rate of 0.001 nm/ps following the procedure in the previous section.

We also performed the same simulation, but instead of starting from the equilibrated system, we placed 10-mer and 5-mer chains randomly in a 9 × 9 × 9 nm^3^ box and repeated the cooling (from 600 to 100 K) and equilibration procedures at each temperature to determine if the starting configurations have any effect on structural properties. We did not observe any major deviations in structure factors, glass transition, or solvent accessible surface area (Figure S10). In separate simulations, we turned our 15-mer polymer chains into 5-mer chains completely by making a second cut in the 10-mer chains (Figure 5). Simulations starting from equilibrated structures as well as from random initial configurations were compared, and no differences were observed in the structural properties (Figure S11).

In order to check the effect of bond scission (10-mer + 5-mer or 5-mer only) on pollutant diffusion, we also performed simulations of damaged polymer chains with 1000 ppm VOCs (acetic acid, formic acid, and formaldehyde) or water. For each simulation, pollutants were inserted into the damaged, bulk polymer equilibrated at 600 K, and then cooled to 100 K in a stepwise manner. After the cooling, an additional 20 ns of equilibration was performed for temperatures 250–500 K using the NPT ensemble. For calculation of diffusion coefficients, we performed a final 10 ns NVT simulation.

### Cross-Linking Simulations

Cross-linking of the side chains may also occurs in acrylic polymers as a result of oxidation and elimination reactions. In order to simulate the effect of cross-linking, we introduced an additional intermolecular “bond” between ester oxygen atoms of acrylic side chains. We first identified pairs of oxygen atoms that are less than 5 Å apart in the equilibrated bulk simulations at each temperature. Only intermolecular cross-linking (between side chains of different polymer chains) was allowed. We also limited each ester oxygen to a single cross-linking bond in cases where multiple oxygen atoms were in the allowed distance range. The identified cross-linking bonds were formed and did not change during the course of the simulation. For each temperature, we simulated the degree of damage by using two different amounts of cross-linking: 5% (75 out of 1500) or 10% (150 out of 1500) of all ester oxygen atoms were cross-linked. In order to form the bond between cross-linking oxygen atoms, we used the “intermolecular interactions” option in Gromacs with simple harmonic potential (function type 1) with bond length of 0.5 nm and constant 327021.44 kJ mol^–1^ nm^–2^, which is the bond constant for the sp^2^ carbon and ester oxygen in GAFF.^36^ After introducing the cross-linking, we repeated the minimization (1000 steps), NVT equilibration (200 ps), and NPT equilibration (10 ns). After this short equilibration, we performed deformation simulations and calculation of stress versus strain curves using straining rate of 0.001 nm/ps as previously mentioned above.

## Results and Discussion

### Glass Transition of Acrylic Copolymers

Before we discuss the different degradation processes and their effects on acrylic polymers, it is necessary to discuss the glass transition behavior of acrylics. The glass transition temperature (*T*_g_) plays a crucial role in acrylic paint formulations because when the paint is soft and rubbery above *T*_g_, its surface becomes too sticky and easily picks up dust, dirt, and other environmental pollutants. On the other hand, at low temperatures below *T*_g_, the glassy acrylic paints face the danger of cracking. For a good acrylic paint, *T*_g_ must be low enough so that the paint film will remain flexible and will not crack but also high enough to prevent the dried film from absorbing pollutants or even being unstable by itself. To have a good balance between glassy and rubbery behavior, acrylic paints are carefully formulated using copolymers of PMMA (hard component) and either PEA or P(nBA) (soft component) to achieve a *T*_g_ close to room temperature (289 K for P(MMA-*co*-EA)^49^ and 280 K for P(MMA-*co*-nBA)^50,51^). Unfortunately, from a practical point of view, having a *T*_g_ close to room temperature requires a good control of temperature and humidity of surroundings and presents challenges to conservators in terms of preventing degradation of artworks. Our work is aimed at providing insight into how temperature and various degradation mechanisms change acrylic polymer structure and properties. To this end, we first determined the glass transition temperature of the acrylic copolymers in our simulations. As discussed in our previous work, due to differences in cooling rates (20 K/ns, which is 1.2 × 10^12^ K/min, in simulations vs 10 K/min in experiments) and molecular weight of polymers (∼1500 Da in simulations), the calculated *T*_g_ values from simulations are higher compared to experiments (414 K for P(MMA-*co*-EA) and 378 K for P(MMA-*co*-nBA)).^34^ In this work, we performed additional simulations with slower cooling rates (10 and 5 K/ns), and our results confirmed that even though *T*_g_ is cooling rate dependent, the change is negligible (Figure S4). The molecular weight of the polymer chains in our model is way below the entanglement lengths for these polymers (*M*_e_ of PMMA = 12500, *M*_e_ of PEA = 7770^52^). An adjustment to the *T*_g_ values using Williams–Landel–Ferry (WLF) equation for linking experimental and simulation cooling rates using values for PMMA (*A* = 17.7 K and *B* = 59.3 K)^53^ gives a correction to glass transition temperature, Δ*T*_g_, of 99.2 K. The corrected *T*_g_ values are 315 K for P(MMA-*co*-EA) and 279 K or P(MMA-*co*-nBA), which suggests our calculated *T*_g_ values are in good agreement with experimentally measured values. This temperature difference of ∼100 K between calculated and measured *T*_g_ should be kept in mind throughout the following discussion. In some cases, we refer to temperatures as above *T*_g_ or below *T*_g_ to emphasize the physical state of the polymers in our simulations at those temperatures.

### Properties of Acrylic Thin Films

Through a combination of physical and chemical processes, aging of modern paints starts from the surface and advances to the inner layers of the paint, where changes in the structure of the polymeric binder cause defects that facilitate the diffusion of small particles.^31^ Because degradation often starts at the surface of the paintings, it is crucial to understand the structure of the exposed surface as well as its interaction with the environment. When a paint is applied onto the canvas, the paint layer can range in thickness between thin paint films (10–50 μm) and much thicker films.^54^ Although it is not computationally feasible to model polymer films of this thickness, we attempt to reproduce the surface structure of the acrylics by constructing thin freestanding films of thickness ∼4 nm. Additional simulations with twice the number of polymer chains, which increased the film thickness to ∼6 nm, revealed that the thickness of the film had no significant effects on the studied properties (see the Methods section for details). This allows us to study the structural changes observed on the surface as well as study VOC absorption and desorption processes that take place. Modeling freestanding polymer films is nontrivial and requires a carefully equilibrated polymer melt.^55^ In our model, the freestanding films were prepared by elongating the simulation box in one direction and exposing the surfaces of the fully equilibrated bulk polymer to vacuum and finally repeating the slow cooling procedure to obtain correct density at each temperature (see the Methods section).

**Figure 1:**
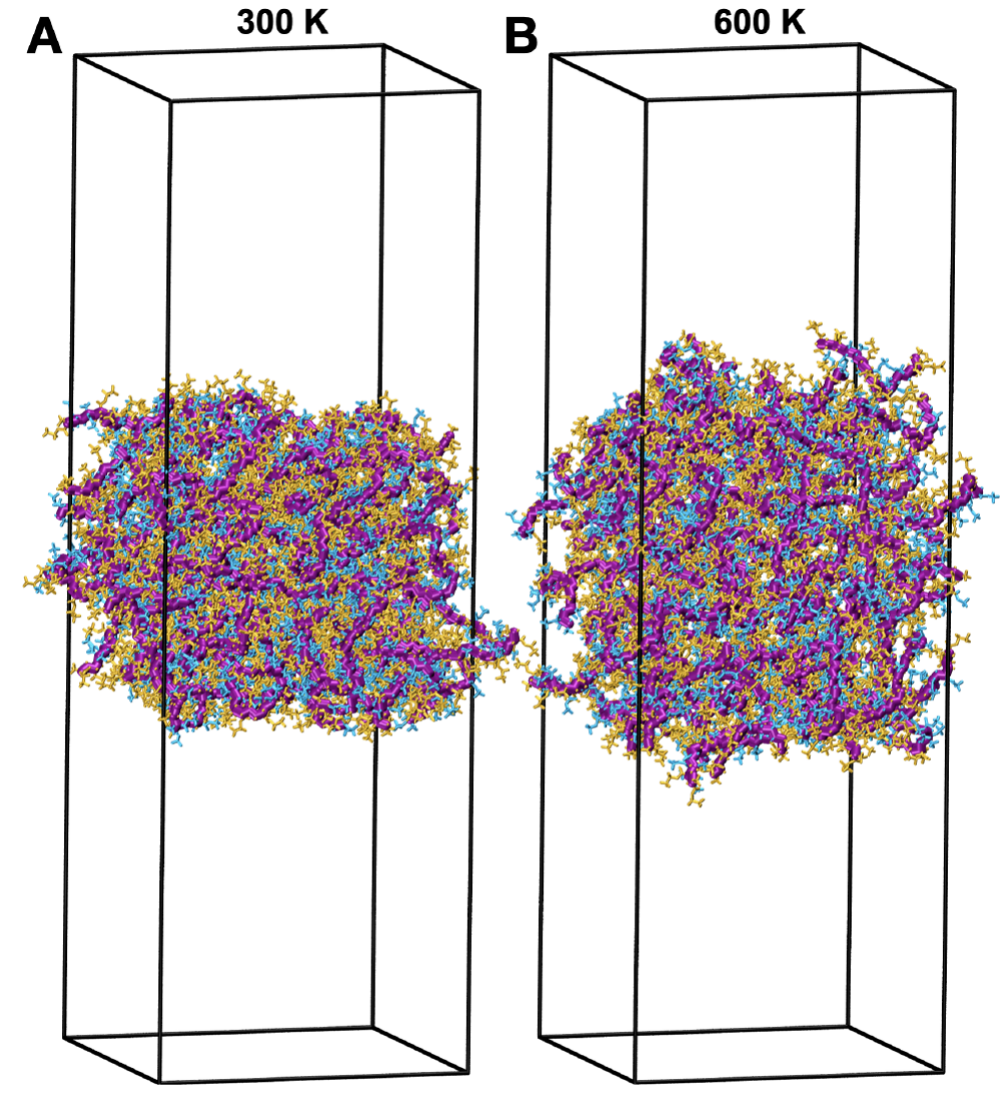
Snapshots of the P(MMA-*co*-EA) films after 100 ns at (A) 300 K and (B) 600 K showing the change in density and thickness of the film. The backbone of the copolymer chains is shown with thick purple lines, MMA monomers in blue, and EA monomers in orange.

The bulk phase^34^ models a continuous polymer in *x*, *y*, and *z* directions using periodic boundary conditions, while the film phase is only continuous in *x* and *y*, producing surfaces in contact with vacuum similar to air on the surface of paintings. Because we performed these simulations under constant temperature and volume (NVT), as the density of the film changes with temperature, we observe a small change in the thickness of the film as shown in the snapshots in Figure 1. Comparison of the film density with the bulk density suggests that the surface effects contribute to a slight decrease in the average density compared to bulk density at high temperatures (Figure S1). We also do not observe a significant difference when we compare the glass transition temperature of the film to the bulk (Figure S12). However, there is an increase in the self-diffusion coefficient of the polymer chains in the film within the accuracy we can compute compared to the bulk phase across the temperature range studied. That can be attributed to the exposed surfaces of the film where the polymer chains freely expand at high temperatures. As a consequence, at high temperatures the difference in diffusion between film and bulk copolymers becomes large. Although below *T*_g_ it is questionable to report diffusion coefficients because the chains have very little or no observable diffusion due to the glassy state, we report apparent diffusion coefficients below *T*_g_ for the sake of comparing polymer motion in bulk and thin film. While the diffusion coefficient of the polymers in the film is ∼2 times faster for both copolymers at 300 K (1.3 × 10^–9^ cm^2^/s (film) versus 6.5 × 10^–10^ cm^2^/s (bulk) for P(MMA-*co*-EA) and 3.3 × 10^–9^ cm^2^/s (film) versus 1.3 × 10^–9^ cm^2^/s (bulk) for P(MMA-*co*-*n*BA)), the increase is 5 times at 600 K. Here we also note the P(MMA-*co*-*n*BA) binder is more mobile than P(MMA-*co*-EA). Other statistical properties of the polymer chains, such as end-to-end distance (*R*_e_) and radius of gyration (*R*_g_), are shown in Figure S13. The end-to-end vector autocorrelation function of the polymers in the film decays quickly to zero above *T*_g_ and shows slow or inhibited relaxation as the temperature decreases. This change in the kinetics of the polymer chains during glass transition consequently affects how environmental pollutants interact with the acrylic films, which will be discussed in the following section.

### Pollutant Absorption from the Environment

**Figure 2:**
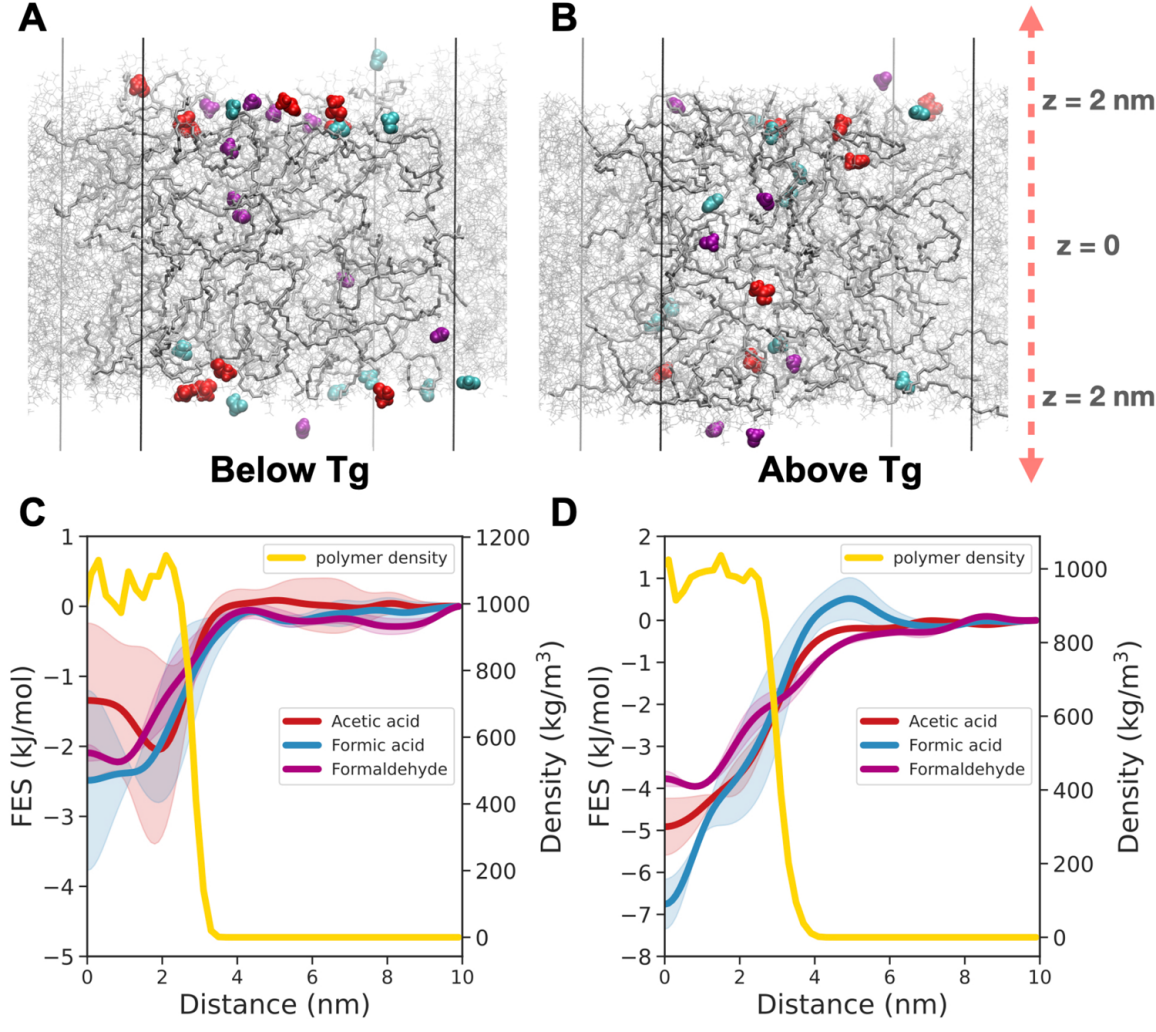
Snapshots of the P(MMA-*co*-EA) films (A) below *T*_g_ (300 K) and (B) above *T*_g_ (450 K). The copolymer chains are shown in gray, acetic acid in red, formic acid in blue, and formaldehyde in purple. The free energy surface (FES) as a function of distance of pollutants from the center of the P(MMA-*co*-EA) polymer film (C) below *T*_g_ and (D) above *T*_g_. The solid lines are averages of two independent metadynamics simulations. The shaded regions show the difference between the two runs. Polymer density (right side of the *y*-axis) is plotted as a function of distance to show where the surface of the film ends with respect to distance.

In order to obtain a molecular picture of how absorption of VOCs or water from the environment into the acrylic paints occurs, we performed thin film copolymer simulations in the presence of acetic acid, formic acid, formaldehyde, or water. Qualitatively, the simulation snapshots in Figure 2A,B reveal that at low temperatures there is only adsorption of VOCs on the film surface, while at high temperatures VOCs are absorbed and easily diffuse inside the film. This is due to several factors. First, the copolymer films are very dense at temperatures below *T*_g_ with small spaces between the chains (Figure S14) that do not allow VOCs to penetrate easily. Furthermore, the diffusion of VOCs in the copolymers is slow at temperatures lower than 400 K,^34^ which makes it difficult for them to move around in the film once they are adsorbed onto the surface. However, as the temperature increases, both the diffusion of VOCs and polymer chains increase as well as a significant increase in the porosity of the copolymer, allowing the molecules to diffuse in and out of the film. We can quantify this observation by calculating the average density of pollutants measured from the center of the film (Figure S15). For all three VOCs, the molecules are concentrated on the surface of the copolymer films, and they slowly start penetrating into the films as the temperature increases. Only at temperatures close to or above *T*_g_ can the VOCs diffuse throughout the entire thickness of the film. Water absorption into the film occurs in a very similar fashion, where at temperatures lower than 450 K, we observe adsorption on the surface and the water molecules penetrate the film at higher temperatures in both acrylic copolymers. In terms of the ability of the pollutants to be absorbed into the film, we notice that the smallest molecules, formaldehyde and water, peak closer to the center of the film compared to the other two below *T*_g_ (Figure S16). At temperatures above *T*_g_, the average densities of acetic acid and formic acid are higher compared to those of formaldehyde and water. This is because formaldehyde and water diffuse considerably faster and are absorbed/emitted into/from the film throughout the simulation time, instead of being stuck between the polymer chains like acetic acid and formic acid molecules.

It is clear from the density profiles of VOCs across the film (Figures S15 and S16) and the snapshots from our trajectories (Figure 2A) that below *T*_g_ the pollutants do not sample the entire thickness of the thin film and are instead adsorbed and stuck on the surface. While above *T*_g_, the distribution of pollutants across the film thickness is more uniform. This behavior is a direct consequence of glass transition. When below a certain temperature, i.e. *T*_g_, dense polymer chains have little or no diffusion and cannot rearrange to allow for absorption and diffusion of pollutants inside the film. Therefore, we hypothesize that there must be a free energy barrier below *T*_g_ that must be overcome for pollutants to be absorbed from the surface further into the polymer film. In addition, some of the pollutants (i.e., acetic acid and formic acid) can become stuck inside the polymer film even above *T*_g_ for long times instead being emitted back into the vacuum. Metadynamics is a powerful enhanced sampling method to study rare events, such as absorption of pollutants at temperatures below *T*_g_, by adding an external history-dependent bias potential in the form of Gaussian potentials deposited along a predefined collective variable (CV) during the simulation. It is important to choose a meaningful CV that is relevant for the process studied. For our case, we chose the *z* component of the distance from center of the polymer film to the center of the VOC. This distance will have a maximum value of 10.0 nm (half the size of the simulation box) and will get closer to zero as the VOC is being absorbed inside the film.

The free energy of absorption for acetic acid, formic acid, and formaldehyde is shown in Figure 2 for 300 K, which is below *T*_g_, and 450 K, which is above *T*_g_. We picked these temperatures to calculate the free energy of absorption of VOCs, because we saw from unbiased simulations that the absorption behavior below *T*_g_ and above *T*_g_ are quite different (Figures S15 and S16). This difference in absorption is also reflected in our calculated free energy surfaces. Above *T*_g_, the polymer chains are mobile and VOCs can diffuse more easily, which allows for sufficient sampling to calculate the free energy surface. This is why we do not see major deviations between the two independent simulations (Figure S17). We should note here that there is no free energy barrier associated with the absorption of pollutants above *T*_g_. The small “barrier” for formic acid in Figure 2D is at a distance of ∼3 nm from the surface of the film and is a result of over sampling of formic acid in the vacuum space between the film surfaces in one of the metadynamics simulations (Figure S7C). On the other hand, below *T*_g_, not only the VOC diffusion is slower, the polymer diffusion is extremely slow due to glassy behavior, which makes it more difficult for the VOC to sample the entire region in the polymer film. We also observe a local minima at a distance of 2 nm from the center of the film, i.e. the surface of the film, which corresponds to the snapshot from our unbiased MD simulations in Figure 2A. As a result, we observe large deviations between the two independent simulations (Figure S18). We should also point out that this difference is more obvious for acetic acid and formic acid compared to formaldehyde, because formaldehyde is smaller and interacts less with the polymer side chains because it lacks hydrogen-bonding interactions. It is clear that above *T*_g_ the interaction of formic acid with the acrylic polymers is stronger than acetic acid and formaldehyde. It faces energy differences of about 7 kJ/mol to escape the film after being absorbed, compared to 5 kJ/mol for acetic acid and 4 kJ/mol for formaldehyde. Nevertheless, *kT* at 300 K is 2.5 kJ/mo,l and these energy barriers are small and can easily be overcome over experimental time scales when the polymer is rubbery at this temperature. Therefore, we would expect to observe frequent absorption and desorption events taking place above the glass transition temperature. This raises the question of whether the absorption and interaction of these pollutants affect the properties of the acrylic paints and contribute to their degradation over long times (i.e., years), which we try to address in the next section.

### Degradation in Acrylics

In general, acrylic polymers are considered to be highly stable, but because they have only been in used in modern paints since the 1900s, there is lack of data concerning their long-term stability for preventive conservation of cultural heritage. Experimental studies on degradation of acrylic paints attempt to predict the state of these materials after hundreds of years by accelerated thermal- or light-induced aging methods.^31,54^ Degradation of polymers can take place as a result of oxidation and elimination reactions, covalent bond scission, or intermolecular cross-linking reactions.^28,56^ When UV light or thermal energy is absorbed by the material, the degradation is initiated by rupture bonds (covalent bond scission) and formation of new bonds (cross-linking). On the other hand, the presence of environmental pollutants, such as VOCs and water, can act as plasticizers and alter their properties that lead to physical degradation. Studies by Chiantore^29,30^ and Smith^54^ showed that the dominant aging mechanism depends on the type of copolymer: chain scission for P(MMA-*co*-EA) and cross-linking for P(MMA-*co*-nBA). In order to quantify degradation, we direct our attention to one of the most studied mechanical properties of acrylic polymers, which is the elastic modulus, by calculating stress versus strain curves from simulations. We consider structural change in terms of three different types of degradation: (1) due to accumulation of environmental pollutants in the acrylics, (2) due to covalent bond scission, and (3) due to cross-linking reactions that may result from exposure to light, temperature changes, chemicals, etc.

**Figure 3:**
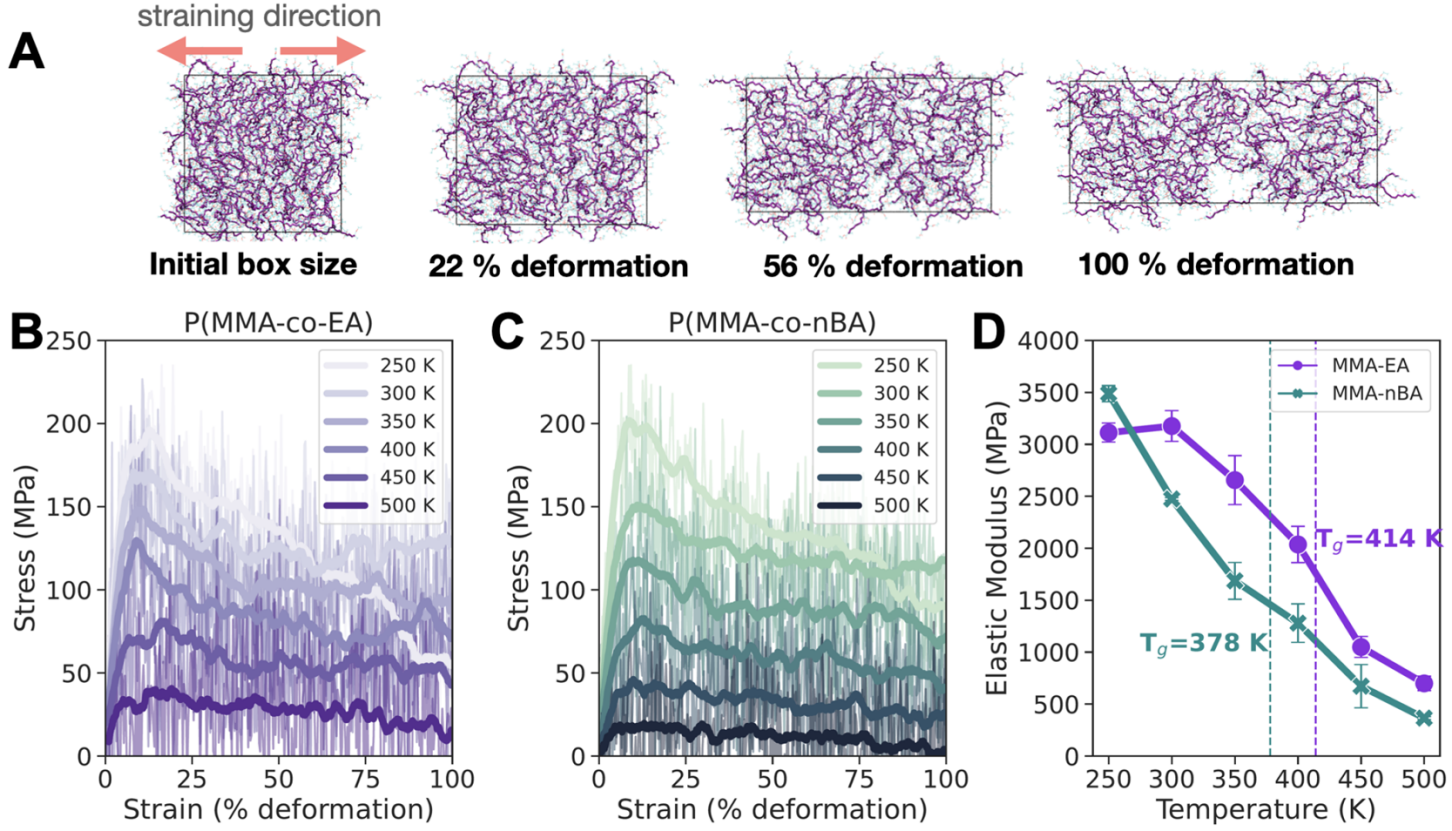
(A) Snapshots obtained at various strain values during uniaxial deformation simulations for P(MMA-*co*-EA) at 300 K. Polymer backbones are shown in purple, side chains in light blue, with oxygen atoms in red. Effect of temperature at constant straining rate of 0.001 nm/ps on stress versus strain curves for (B) P(MMA-*co*-EA) and (C) P(MMA-*co*-nBA). The fluctuations in the stress are shown in the background, and a running average for each temperature is plotted as thick lines for better visualization. (D) Elastic modulus calculated for the regions when strain is less than 4% as a function of temperature. The error bars are standard error of mean calculated from elastic modulus values for deformations along *x*, *y*, or *z* directions.

In order to establish a control for comparison, we first calculate the elastic modulus of P(MMA-*co*-EA) and P(MMA-*co*-nBA) as a function of temperature. The equilibrated bulk copolymers are subjected to uniaxial deformation using a constant straining rate (Figure 3A). The stress–strain curves are obtained as averages from deformations in *x*, *y*, and *z* directions (see the Methods section for detailed information). There are multiple factors that affect the resulting stress versus strain curves obtained in MD simulations, two of which are straining rate (Figure S19) and temperature (Figure 3). If the sample is strained too fast (0.1 nm/ps), then there is not enough sampling for the elastic region before the material reaches its yield point. This makes it difficult to calculate properties such as the elastic modulus. While a slow deformation (0.001 nm/ps) takes a longer time from a computational perspective, more data can be obtained for both elastic and plastic regions of the curves. We should mention that because stress (σ) is calculated from the pressure tensor, at this straining rate we observe large fluctuations in the stress that make it difficult to observe the stress–strain behavior clearly. Therefore, we also plotted a running average of every 200 ps in Figure 3B,C. The effect of temperature is similar for both polymers. At low temperatures we observe the highest yield strengths, and it decreases as the temperature increases, meaning the material transitions from glassy state to a softer, rubbery state. This change in mechanical properties in response to temperature is best represented by calculating the elastic modulus (Figure 3D), which can be compared to experimental values found in the literature. The elastic modulus is calculated from the slope of stress versus strain curves in the elastic region, where the relationship between stress and strain is linear. It can be challenging to define this region because it highly depends on the strain rate and the strain value that is used. We calculated elastic modulus values as a function of temperature using the linear region up to 4% strain (Figure S20). Further deformation of the polymer to 100% strain causes brittle failure below *T*_g_*,* as can be seen in the last snapshot of Figure 3A. For both copolymers, the effect of temperature on elastic modulus is similar, where we see a decrease in modulus with temperature (material becomes softer). We also show that changes in the elastic modulus match with glass transition temperature as expected. For amorphous polymers, fully glassy behavior gives a modulus of ∼3000 MPa, and the transition to rubbery state begins around ∼2000 MPa.^57,58^ Our calculated elastic modulus is in good agreement with this observation as we also see similar values for the transition from the glassy to rubbery region.

When the two copolymers are compared, we observe that P(MMA-*co*-nBA) is softer compared to P(MMA-*co*-EA). Early acrylic paint formulations consisted of a random copolymer of MMA and EA, where MMA (∼40%) was used as the hardening component and EA (∼60%) as the softening component. During the 1950s, the paint manufacturers began using *n*BA monomers in place of EA to lower *T*_g_ even further. The low elastic modulus of P(MMA-*co*-nBA) makes this material easier to work with. There are many studies in the literature concerning different environmental effects, such as temperature, relative humidity, and aging, on mechanical properties of acrylic paints.^59^ It is difficult to compare our calculated elastic modulus values to the ones published in the literature because of variations in the samples (aging, relative humidity, temperature, or polymer and pigment compositions) and experimental methods (straining rates or calculation of modulus) highly affect the reported values.^59^ However, our values agree well with some of the values in the literature. For example, Ormsby and Verfasser^60^ reported a modulus of 3300 MPa at 21 °C (20% RH) for Liquitex brand acrylics with composition P(MMA-*co*-nBA). In comparison we calculated a modulus of 2772 MPa (300 K, 27 °C) for just the P(MMA-*co*-nBA) copolymer with no water or additives. In another study, where different acrylic copolymer binders were compared, the reported modulus values for P(MMA-*co*-EA) are higher in comparison to those for P(MMA-*co*-nBA),^58^ also in agreement with the trends we observe.

### Degradation by Absorption of Environmental Pollutants

**Figure 4:**
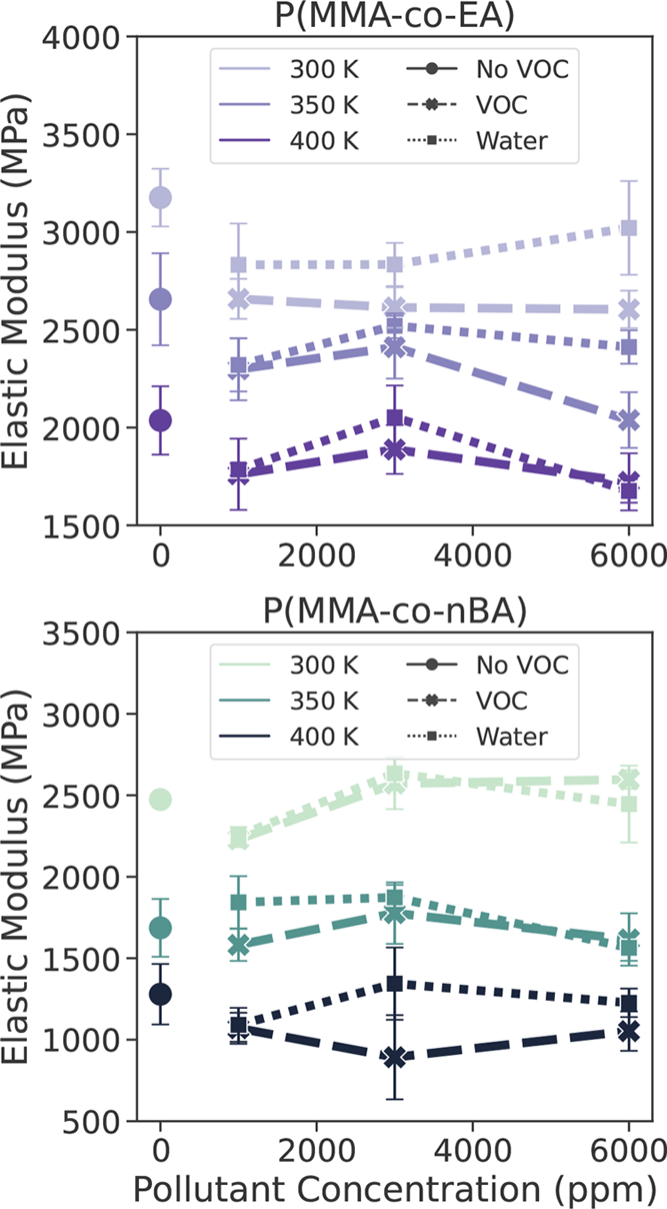
Elastic modulus P(MMA-*co*-EA) and P(MMA-*co*-nBA) as a function of pollutant concentrations in ppm at 300, 350, and 400 K with strain rate of 0.001 nm/ps at strain values up to 4%. Here VOC refers to acetic acid, formic acid, and formaldehyde. The error bars are standard error of mean calculated from elastic modulus values for deformation along *x*, *y*, or *z* directions.

Earlier we focused on the absorption of pollutants from the environment into acrylics; here we would like to focus on how the interaction between environmental pollutants affects the properties of the acrylic polymers. We performed derformation simulations for acrylic copolymers with 1000, 3000, and 6000 ppm concentrations of acetic acid, formic acid, formaldehyde, or water at 300, 350, and 400 K in order to study whether the presence of VOCs or increase in relative humidity changes mechanical properties of acrylics. These VOC concentrations are much higher than measured amounts in museums because very small concentrations would require very large system sizes, which is not possible to simulate with atomistic models. In museums, measured values of pollutants can differ depending on museum environment, display cases, and enclosures. Typical measured concentrations are 83–822, 33–276, and 10–700 ppb for acetic acid, formic acid, and formaldehyde, respectively.^61^ However, in some cases, concentrations as high as 4600 ppb acetic acid were measured.^62^ Typical relative humidity values in a museum gallery are 15–20% during winter and 60–65% during summer.^61^ We chose temperatures around the glass transition temperatures of the acrylic copolymers calculated from simulations (414 and 378 K for P(MMA-*co*-EA) and P(MMA-*co*-nBA), respectively). For temperatures too far above glass transition (>450 K), both of the acrylic copolymers are already very soft and show low modulus values typical of Vogel–Fulcher behavior (Figure 3D), which would make it difficult to observe if the presence of pollutants changes the mechanical properties. At very low temperatures (<300 K), the acrylics are very stiff, making it less probable for pollutants to be absorbed and diffuse inside the polymer. The effect of relative humidity (water content) is well understood for acrylic paints in the literature.^20,33,63^ As the relative humidity increases the modulus decreases in acrylic paints, making the material softer. However, to our knowledge there are no studies on the effect of VOCs on mechanical properties of acrylic paints. Figures S21 and S22 show the full and elastic region of stress versus strain curves at 300 K for the two copolymers studied as a function of pollutant concentration. Although the changes in stress versus strain are not very clear when the full profile is considered, we see a small change in the slope of the lines when looking at the elastic region. Figure 4 suggests that the effect of pollutants depends on the copolymer as well as the temperature. As we can see in this figure, the presence of pollutants (VOCs or water) decreases the modulus for the copolymer P(MMA-*co*-EA) at all temperatures. This change in modulus varies slightly for different concentrations of pollutants, but considering the error bars the effect of concentration is not significant. Especially for P(MMA-*co*-EA), we see the plasticizer effect of the VOCs as the presence of these pollutants makes the material softer. For P(MMA-*co*-nBA), the elastic modulus is more or less unaffected by the presence of pollutants. Although we observe some increase or decrease with concentration and temperature, the change from the original value without any pollutants is rather small when the error bars are considered. Because P(MMA-*co*-nBA) is already a softer material compared to P(MMA-*co*-EA), it is possible that introduction of pollutants does not significantly change its ability to resist deformation. More experimental data concerning pollutant effects on mechanical properties are needed in order to make better connections and to interpret our simulation results.

### Degradation by Bond Scission

**Figure 5:**
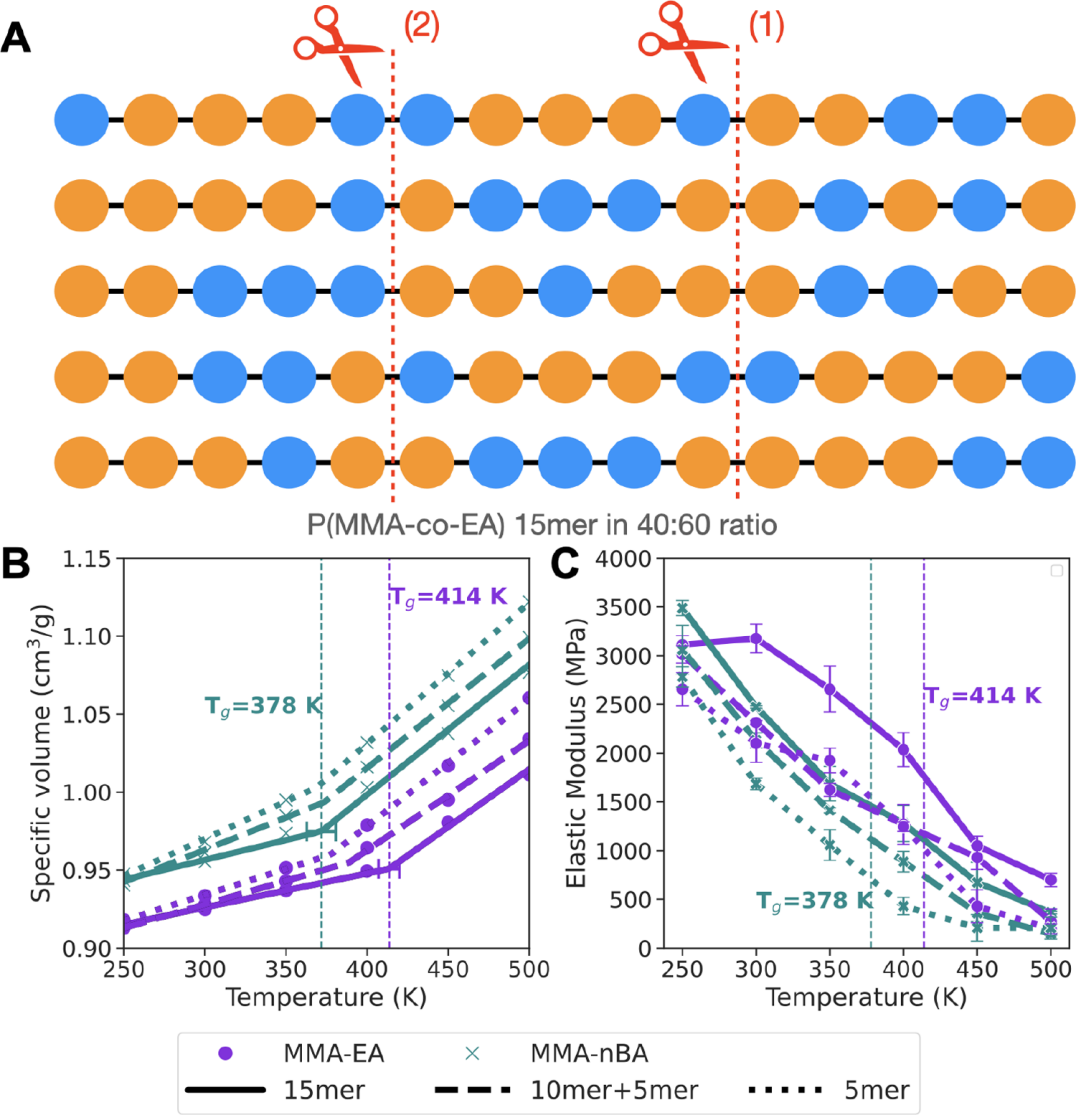
(A) Five different copolymer chain sequences used in the simulations, where blue spheres represent MMA and orange spheres represent EA (or nBA). For the bond scission simulations, we make a cut at point (1), which makes 10-mer + 5-mer chains. In the second step, we make another cut at point (2), which results in all of the chains to be 5-mers. (B) Change in specific volume with temperature, showing the glass transition temperature, *T*_g_, calculated previously for the original copolymers. The error bars are nonlinear standard error associated with the piecewise linear fitting.^64^ (C) Elastic modulus is calculated for P(MMA-*co*-EA) and P(MMA-*co*-nBA) as a function of temperature with strain rate of 0.001 nm/ps at strain values up to 4%. Solid lines refer to undamaged 15-mer copolymer chains, dashed lines are after first bond scission (10-mer + 5-mer), and dotted lines are after the second bond scission (5-mer only). The error bars are the standard error of mean calculated from elastic modulus values for deformation along *x*, *y*, or *z* directions.

As mentioned earlier, degradation can also take place in acrylic paints due to rupture of the carbon–carbon polymer backbone bonds (bond scission) when the materials are exposed to temperature or UV light for long periods of time. This results in a decrease in the molecular weight of the polymer, and the formation of small fragments can not only decrease the mechanical strength of the material but also further attract pollutants from the environment.^31^ Simulation of such chemical reactions involving fragmentation (or cross-linking) requires breaking (or formation) of bonds, which is not possible with nonreactive force fields and also would require much longer simulations. Therefore, we simplified the bond scission reaction by manually breaking the bonds in the polymer backbone. In order to simulate the effect of this type of degradation, we performed bulk simulations where we used our polymer model made up of 15 monomers for each polymer chain and breaking the chains into smaller fragments in two steps: 10-mer + 5-mer and only 5-mer. The acrylic binders used by paint manufacturers are random copolymers of 40% MMA and 60% EA (or nBA). Therefore, we originally prepared our model so that we have five different 15-mer chains in our simulation box (20 of each chain, making a total of 100 polymer chains). Each chain is a combination of MMA and EA (or nBA) with a 40:60 ratio. We introduce the bond scission by breaking the chemical bonds between the 10th and 11th monomer on each chain, resulting in a system with 10-mer chains and 5-mer chains (100 polymer chains of each). In the next step of the bond scission, we cut another bond in order to make 10-mer chains into 5-mer chains, resulting in only 5-mer chains (300 polymer chains in total). This process is shown in Figure 5.

The damage that occurs by breaking of the bonds (through absorption of either heat or light) is expected to result in structural changes that affect the materials properties of the acrylics over time. We calculated stress versus strain curves by performing deformation simulations on the damaged systems (Figure S23). We can see the strong dependence on temperature of elastic moduli from these curves. The elastic modulus values calculated from the slope of the elastic region in Figure 5 shows the effect of damage on the acrylic material properties: as the polymer chains are damaged further, the material loses its stiffness and becomes softer. In this case, the fragmentation of polymer chains leads to smaller molecular weight polymers that can act as plasticizers inside the bulk polymer and aid the overall loss in mechanical strength observed with damage. We also see a small change in glass transition temperature for P(MMA-*co*-EA) (Figure 5), where the damaged chains show a decrease in *T*_g_. This effect, however, is not observed for P(MMA-*co*-nBA). For both copolymers, the bond scission reaction also increases diffusion of the polymer chains (Figure S24). However, we do not observe a significant change in the structure factors (Figure S25). This shows that these small changes in the structure as a result of degradation, such as bond breaking, do not alter the packing of the polymer chains or the volume of the simulation box, as much as the response of the material to deformation in terms of modulus or diffusion of the polymer chains. Thus, measuring mechanical properties in experiments or simulations is more useful than other structural properties in terms of understanding the changes that occur with degradation of acrylics.

### Degradation by Intermolecular Cross-Linking

**Figure 6:**
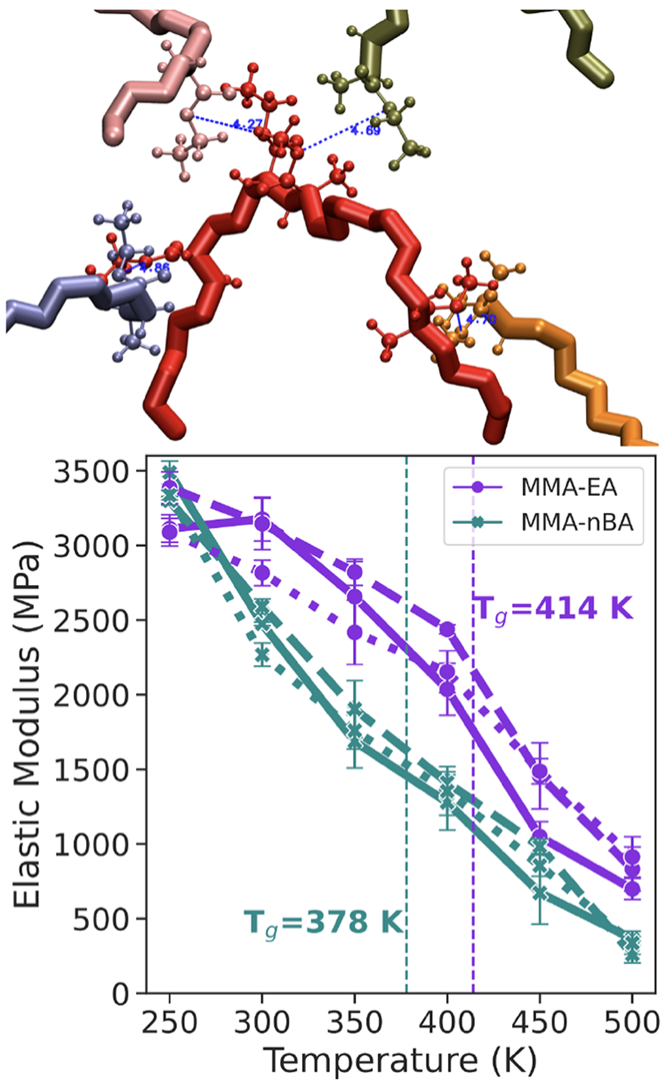
Intermolecular cross-linking between a polymer chains and its effect on elastic modulus. A cross-link can form only if the two ester oxygen atoms are less than 5 Å apart (shown in blue). A single polymer chain (shown in red) can make multiple cross-links with other polymer chains. Elastic modulus is calculated for P(MMA-*co*-EA) and P(MMA-*co*-nBA) as a function of temperature with strain rate of 0.001 nm/ps at strain values up to 4%. Solid lines (**—**) refer to 15-mer copolymer chains with no cross-linking; dotted lines (···) refer to 5% and dashed lines (---) refer to 10% of cross-linking between ester oxygens. The error bars are standard error of mean calculated from elastic modulus values for deformation along *x*, *y*, or *z* directions. See Figure S26 for stress versus strain curves.

Another degradation process that commonly takes place in acrylics is the cross-linking of the polymer chains as a result of oxidation reactions of the ester groups on the polymer side chains. Degradation by cross-linking reactions was observed to be the dominant mechanism in acrylic polymers that have longer alkyl side chains, such as P(nBA).^29,30^ The oxidation of the side chains may lead to decomposition and loss of the butyl groups. At the same time, the high mobility and flexibility of the P(nBA) side chain facilitate the formation of cross-links to produce a polymer network. When cross-linking is the dominant mechanism of degradation in acrylics, it displays an opposite effect to bond scission reactions in terms of the material mechanical properties, where thermal mechanical analysis measurements showed an increase in softening temperature.^30^ As in the case of bond scission, we used a simple approach to simulate the cross-linking that occurs as a result of the oxidation reaction by manually making new bonds between the side chains of the polymer. For our simple cross-linking scheme, the ester oxygen on each monomer was allowed to make only a single intermolecular cross-link with another ester oxygen if the distance between the two oxygen atoms was less than 5 Å, shown in Figure 6. We identified the cross-links from equilibrated structures at each temperature, and once the cross-link was made, this new bond was kept stable throughout the simulation using a harmonic potential (see the Methods section for details). It should be noted that in order to separate the effects of cross-linking from bond scission, the cross-linking process in our simulations does not involve the fragmentation and loss of the alkyl side chain that is observed in experiments.

At a first glance, the intermolcular cross-linking we attempted to simulate in this simple way does not show considerable differences in the properties of the acrylics. The glass transition temperature and the self-diffusion coefficient of the polymer chains remain unaffected (Figure S27). In terms of the effect that intermolecular cross-linking has on the mechanical properties of acrylic paints, we observe small changes in elastic modulus that is mostly within the error bars. The acrylic polymers become slightly harder with more cross-linking, which is more apparent for temperatures near or above the glass transition when the polymer is soft and rubbery. In our simulations, a maximum of 10% of polymer side chains participate in cross-linking interactions in order to be consistent with the amount of cross-linking throughout the entire temperature range. This is because even though at lower temperatures the polymer chains are more densely packed and more cross-linking is possible, at high temperatures the polymer chains are not in close proximity to allow for these bonded interactions. Furthermore, experiments lack information about the amount of cross-linking reactions that occur as acrylic polymers age. From our simulations, we would expect that with higher amount of cross-linking these acrylics will become harder.

### Pollutant Diffusion in Degraded Polymers

We are also interested in how chemical damage affects the interaction of environmental pollutants with copolymers. We showed that while cross-linking reactions lead to slight hardening, bond scission reactions in acrylic copolymer result in softening of the material. Fragmentation of the copolymer chains through bond scission also causes a decrease in the density of the material (Figure 5), which will allow the pollutants from the environment to be more easily absorbed compared to undamaged or cross-linked polymers. Here, in an effort to explore the combined effect of chemical damage and interactions of pollutants with the acrylic polymers, we performed simulations of degraded polymers with 1000 ppm concentration of pollutants.

**Figure 7:**
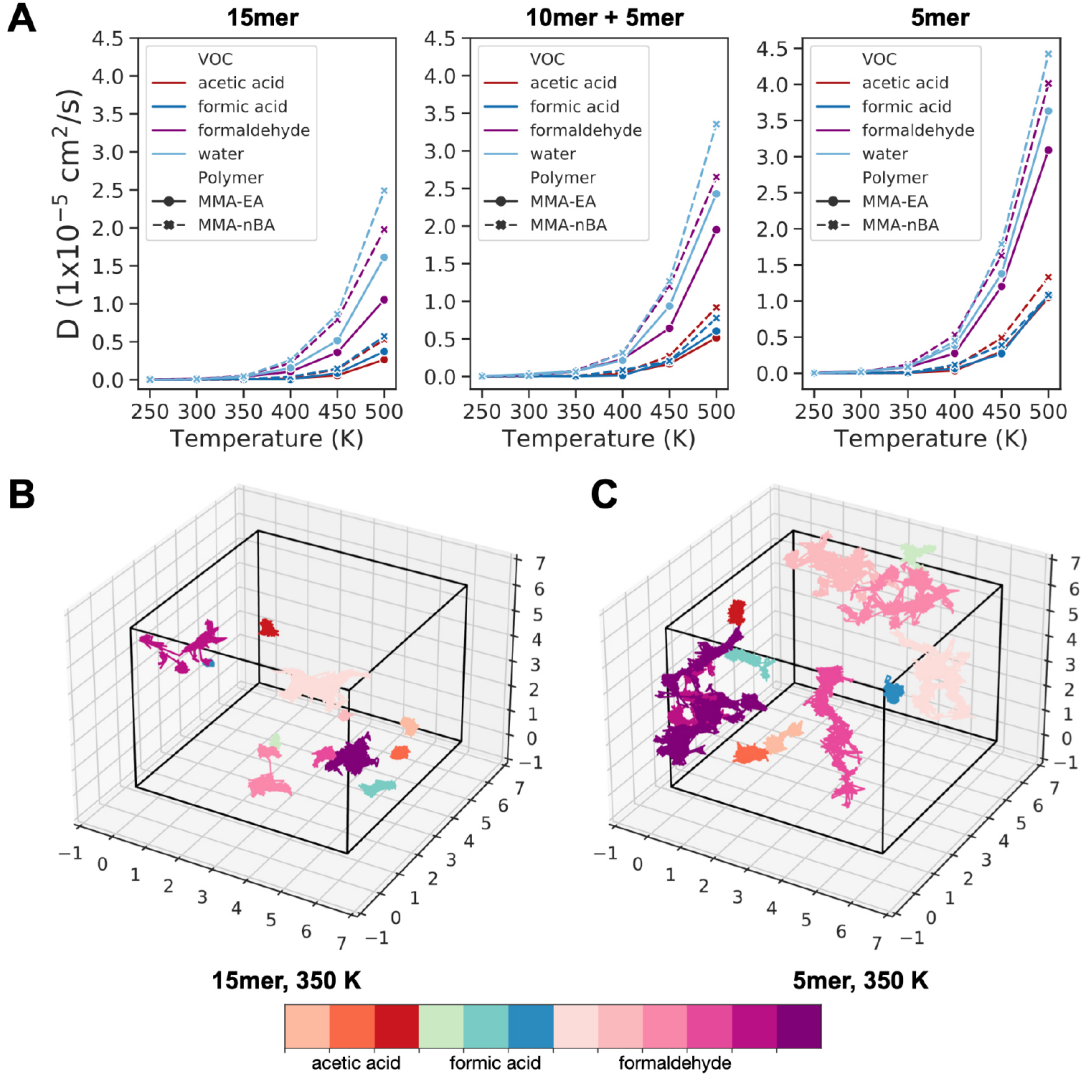
(A) Self-diffusion coefficients of 1000 ppm concentration of pollutants in undamaged (15-mer), partially damaged (10-mer + 5-mer), and fully damaged (5-mer) P(MMA-*co*-EA) and P(MMA-*co*-nBA) as a function of temperature. Corresponding center-of-mass trajectories of VOCs at 350 K in (B) undamaged 15-mer and (C) damaged (5-mer) P(MMA-*co*-EA).

We calculated diffusion coefficients of pollutants by calculating the mean square displacement (MSD) over time for each pollutant molecule and using the diffusive region where the slope is close to 1.0 to calculate the self-diffusion coefficients for each pollutant as a function of temperature (Figure S28). In the same way, the diffusion coefficients of the damaged polymer chains are calculated. The relationship between diffusion coefficients of polymer chains, pollutants and temperature with bond scission damage is shown in Figures 7 and S29. The trends we observed previously in undamaged systems are still valid, where the diffusion coefficients follow the trend water > formaldehyde > formic acid > acetic acid in both copolymers. There is an increase in the diffusion coefficients of pollutants as the copolymer chains are broken. The diffusion coefficient for each pollutant follows the trend undamaged (15-mer) < after first bond scission (10-mer + 5-mer) < after second bond scission (5-mer only), meaning the shorter polymer chains allow for pollutants to diffuse faster (Figure 7). Not only is the diffusion of pollutants enhanced with damage, the diffusion of the polymer chains is also faster in the damaged systems (Figure S24).

Finally, we can visualize the diffusion of pollutants in the damaged copolymer compared to diffusion in the undamaged copolymer, shown in Figure 7B,C. It is clear that when the polymer chains are shorter (damaged), VOCs diffuse more in the simulation box over time. This is true for low temperatures (Figure S30), where previously our results showed little or no diffusion, as well as temperatures above *T*_g_ (Figure S31). We also observed the same behavior for water (Figures S32–S34). The diffusion mechanism is similar to what is observed before, where particles are trapped and diffuse through small oscillating motions in local cages for a long period of time before they jump into a new cage. However, because the polymer chains are shorter, it is much easier for the polymers to rearrange and open these empty spaces for the pollutants. This observation suggests that we would encounter more instances of absorption of pollutants from the surrounding environment into acrylics when they are damaged via bond scission reactions.

## Conclusions

There are many different mechanisms of degradation in acrylic polymers that are commonly known. In addition to commonly observed chemical degradation mechanisms, such as bond scission and cross-linking reactions, absorption of pollutants from the environment also poses significant risks to their longevity. In this work, we developed a computational model to understand how these degradation mechanisms change material properties of the two most commonly used copolymers in acrylic paints. It is known from experiments and visual inspection of artwork that the surface of acrylic paints can become soft above the glass transition temperature and attract pollutants and dust from their environment. From a conservator’s perspective, this may cause coloration of binder and undesirable visual defects on the painting surface. We study this process in our atomistic simulations by exposing thin polymer films to most abundant pollutants, such as acetic acid, formic acid, formaldehyde, and water, at different temperatures. Because the glass transition temperature of these acrylic polymers is close to room temperature, we observe significant differences in pollutant absorption depending on whether the polymer is in glassy or rubbery state. The free energy of absorption for all of the pollutants is favorable (−4 to −7 kJ/mol) above the glass transition temperature, suggesting that pollutants tend to be absorbed into the acrylic polymers and may be trapped between the polymer chains. These pollutants when absorbed in the material cause a decrease in elastic modulus, especially in P(MMA-*co*-EA), acting as plasticizers in the polymer material.

Other types of chemical degradation, such as bond scission reactions and cross-linking reactions, are more difficult to simulate with molecular dynamics simulations using nonreactive, classical force fields. However, because these chemical reactions in acrylic polymers are well-studied in the literature and the products are known, we modeled the polymers that result from these chemical degradation processes using simpler methods. Our bond scission simulations, where polymer chains are broken along their backbone gradually, suggest that this type of degradation results in significant changes in the mechanical properties of the acrylics. In addition to the elastic modulus, other microscopic properties, such as diffusion and density of polymer chains, are also affected by the length of the polymer chains. The fragmentation of the polymer backbones makes the material softer and less resistant to deformation. In addition, an increase in the polymer chain diffusion allows for faster diffusion of pollutants once they are absorbed in the material. This, along with the plasticizer effect of environmental pollutants, will further cause softening and loss of stability. On the other hand, our model of the intermolecular cross-linking reactions, which is another type of chemical degradation mechanism commonly observed in acrylics, did not reveal any insights into how this reaction changes the microscopic properties of acrylics. This may be due to limitations in our model, the low amount of cross-linking introduced, or the properties calculated. The results from our simulations should contribute to filling the gap in knowledge between microscopic, structural changes in acrylic polymers and degradation processes that are observed in practice.

## Supporting Information Available

A table of all simulations performed in this work, supporting figures for the methods and results sections on effect of cooling rates, system size effects, metadynamics simulations, polymer thin film properties, pollutant absorption, stress vs strain curves (PDF)
