# Supplementary material for: Molecular Simulation Strategies for Understanding the Degradation Mechanisms of Acrylic Polymers
Source: Macromolecules. 2023 Apr 19;56(9):3272–85. doi: 10.1021/acs.macromol.2c02442 (PMC10174159; doi:10.1021/acs.macromol.2c02442)
Supplement: Supplementary file 1 — ma2c02442_si_001.pdf [file ma2c02442_si_001.pdf]

# Supporting Information: Molecular Simulation Strategies for Understanding the Degradation Mechanisms of Acrylic Polymers

Aysenur Iscen,[†] Nancy C. Forero-Martinez,[†] Omar Valsson,[†,‡] and Kurt Kremer[*,†]

†*Max Planck Institute for Polymer Research, Ackermannweg 10, 55128 Mainz, Germany*

‡*Department of Chemistry, University of North Texas, Denton, TX, USA*

E-mail: kremer@mpip-mainz.mpg.de

# Supporting information for Methods

Table S1: Summary of all the simulations performed in this study. The total number of atoms is given for P(MMA-co-EA). For simulations with P(MMA-co-nBA) and no additional components, the number of atoms is 28700. All of the simulations amounts to ∼11 µs. Minimization and short NVT equilibration steps are not listed in the table below.

| Copolymer | Polymer size | Additional system components | Total number of atoms | Description | Simulation steps | Simulated temperatures | Total simulation Time (ns) |
|---|---|---|---|---|---|---|---|
| P(MMA-co-EA) or P(MMA-co-nBA) | 100 polymer chains (15mer) | None | 23300 | Bulk polymer, deformation simulation (0.001 nm/ps) | 8 ns NPT × 3 independent runs for x, y, z deformation | 250, 300, 350, 400, 450, 500 K | 288 |
| | | | | Bulk polymer, deformation simulation (0.01 nm/ps) | 8 ns NPT ×3 independent runs for x, y, z deformation | 300 K | 48 |
| | | | | Bulk polymer, deformation simulation (0.1 nm/ps) | 8 ns NPT × 3 independent runs for x, y, z deformation | 300 K | 48 |
| | | | | Bulk polymer, cross-linking | 10 ns NPT, 8 ns NPT × 3 independent runs for x, y, z deformation × 2 degrees of cross-linking (5 %, 10 %) | 250, 300, 350, 400, 450, 500 K | 288 |
| | | 3 acetic acid, 3 formic acid, 6 formaldehyde | 23363 | Bulk polymer + 1000 ppm VOCs, deformation simulation (0.001 nm/ps) | 8 ns NPT × 3 independent runs for x, y, z deformation | 300, 350, 400 K | 144 |
| | | 9 acetic acid, 12 formic acid, 18 formaldehyde | 23504 | Bulk polymer + 3000 ppm VOCs, deformation simulation (0.001 nm/ps) | 8 ns NPT × 3 independent runs for x, y, z deformation | 300, 350, 400 K | 144 |
| | | 18 acetic acid, 24 formic acid, 36 formaldehyde | 23708 | Bulk polymer + 6000 ppm VOCs, deformation simulation (0.001 nm/ps) | 8 ns NPT × 3 independent runs for x, y, z deformation | 300, 350, 400 K | 144 |

| | | | | | | | |
|---|---|---|---|---|---|---|---|
| | | 12 water | 23336 | Bulk polymer + 1000 ppm water, deformation simulation (0.001 nm/ps) | 8 ns NPT × 3 independent runs for x, y, z deformation | 300, 350, 400 K | 144 |
| | | 30 water | 23390 | Bulk polymer + 3000 ppm water, deformation simulation (0.001 nm/ps) | 8 ns NPT × 3 independent runs for x, y, z deformation | 300, 350, 400 K | 144 |
| | | 60 water | 23480 | Bulk polymer + 6000 ppm water, deformation simulation (0.001 nm/ps) | 8 ns NPT × 3 independent runs for x, y, z deformation | 300, 350, 400 K | 144 |
| | | None | 23300 | Thin film | 25 ns NVT cooling (600 to 100 K), 100 ns NVT equilibration at each temperature | 100, 150, 200, 250, 300, 350, 400, 450, 500, 550, 600 K | 2250 |
| | | 10 acetic acid, 10 formic acid, 10 formaldehyde | 23470 | Thin film + VOCs | 10 ns NVT | 250, 300, 350, 400, 450, 500 K | 120 |
| | | 30 water | 23390 | Thin film + water | 10 ns NVT | 250, 300, 350, 400, 450, 500 K | 120 |
| P(MMA-co-EA) only | | None | 23300 | Thin film, effect of cooling rate | NVT cooling (10 K/ns and 5 K/ns) | 600 to 100 K | 150 |
| | | 1 acetic acid | 23308 | Thin film + VOC, metadynamics | 100 ns NVT × 2 independent runs | 300, 450 K | 400 |
| | | 1 formic acid | 23305 | Thin film + VOC, metadynamics | 100 ns NVT × 2 independent runs | 300, 450 K | 400 |
| | | 1 formaldehyde | 23304 | Thin film + VOC, metadynamics | 100 ns NVT × 2 independent runs | 300, 450 K | 400 |
| P(MMA-co-EA) or P(MMA-co-nBA) | 200 polymer chains (50 % 10mer + 50 % 5mer) | None | 23300 | Bulk polymer, bond scission | 25 ns NPT cooling (600 to 100 K), 20 ns NPT equilibration × 2 (starting from equilibrated or random chains) | 100, 150, 200, 250, 300, 350, 400, 450, 500, 550, 600 K | 980 |
| | | | | Bulk polymer with bond scission, deformation simulation (0.001 nm/ps) | 8 ns NPT × 3 independent runs for x, y, z deformation | 250, 300, 350, 400, 450, 500 K | 288 |

| | | | | | | | |
|---|---|---|---|---|---|---|---|
| | | 3 acetic acid, 3 formic acid, 6 formaldehyde | 23363 | Bulk polymer with bond scission + 1000 ppm VOCs | 25 ns NPT cooling (600 to 100 K), 20 ns NPT equilibration, 10 ns NVT (for diffusion calculation) | 250, 300, 350, 400, 450, 500 K | 410 |
| | | 12 water | 23336 | Bulk polymer with bond scission + 1000 ppm water | 25 ns NPT cooling (600 to 100 K), 20 ns NPT equilibration, 10 ns NVT (for diffusion calculation) | 250, 300, 350, 400, 450, 500 K | 410 |
| | 300 polymer chains (5mer) | None | 23300 | Bulk polymer, bond scission | 25 ns NPT cooling (600 to 100 K), 20 ns NPT equilibration × 2 (starting from equilibrated or random chains) | 100, 150, 200, 250, 300, 350, 400, 450, 500, 550, 600 K | 980 |
| | | | | Bulk polymer with bond scission, deformation simulation (0.001 nm/ps) | 8 ns NPT × 3 independent runs for x, y, z deformation | 250, 300, 350, 400, 450, 500 K | 288 |
| | | 3 acetic acid, 3 formic acid, 6 formaldehyde | 23363 | Bulk polymer with bond scission + 1000 ppm VOCs | 25 ns NPT cooling (600 to 100 K), 20 ns NPT equilibration, 10 ns NVT (for diffusion calculation) | 250, 300, 350, 400, 450, 500 K | 410 |
| | | 12 water | 23336 | Bulk polymer with bond scission + 1000 ppm water | 25 ns NPT cooling (600 to 100 K), 20 ns NPT equilibration, 10 ns NVT (for diffusion calculation) | 250, 300, 350, 400, 450, 500 K | 410 |
| P(MMA-co-EA) only | 200 polymer chains (15mer) | None | 46600 | Bulk polymer, system size effects | 25 ns NPT cooling (600 to 100 K), 100 ns NPT equilibration at each temperature | 100, 150, 200, 250, 300, 350, 400, 450, 500, 550, 600 K | 1125 |
| | | | | Thin film, system size effects | 25 ns NVT cooling (600 to 100 K), 100 ns NVT equilibration at each temperature | 300, 450 K | 225 |
| | | 10 acetic acid, 10 formic acid, 10 formaldehyde | 46770 | Thin film with VOCs, system size effects | 10 ns NVT | 300, 450 K | 20 |

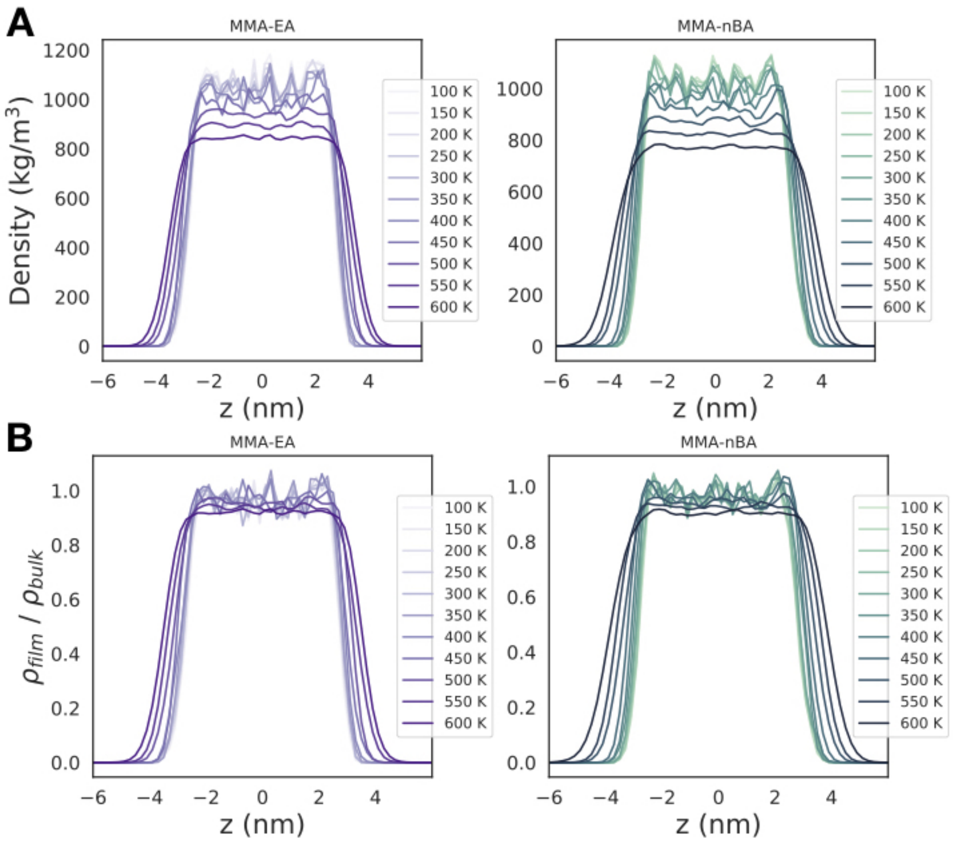


Figure S1: (A) Average density and (B) density ratio with respect to the bulk phase of the polymer chains along the z-axis (thickness) of the film calculated for P(MMA-co-EA) and P(MMA-co-*n*BA) at different temperatures.

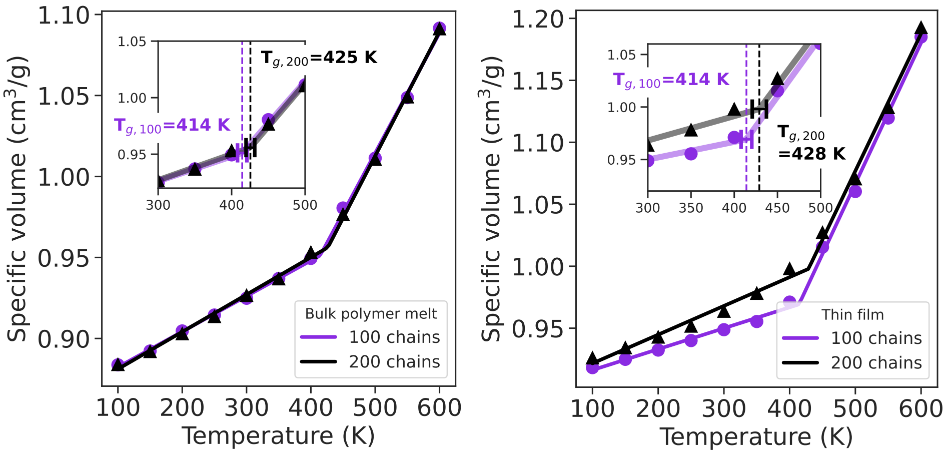


(a) Bulk polymer (b) Thin film

Figure S2: Effect of system size on glass transition temperature of bulk and thin film P(MMA-co-EA) polymer chains. The error bars are non-linear standard error associated with the piece-wise linear fitting.

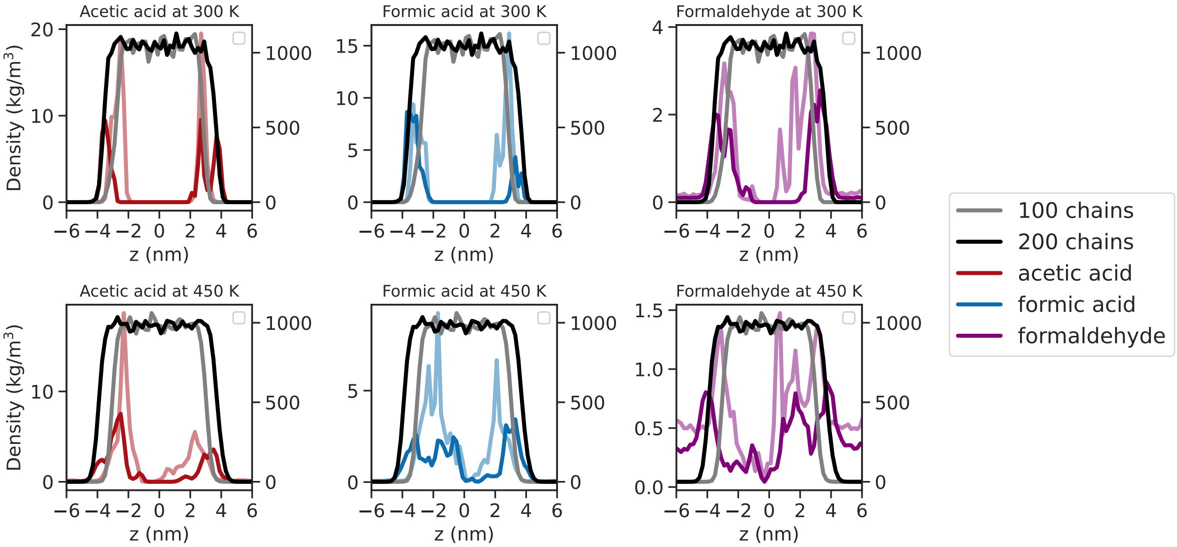


Figure S3: Effect of film thickness on glass transition temperature and pollutant density distribution across the film. Bold colors correspond to the system with 200 polymer chains, while light colors correspond to smaller system with 100 polymer chains.

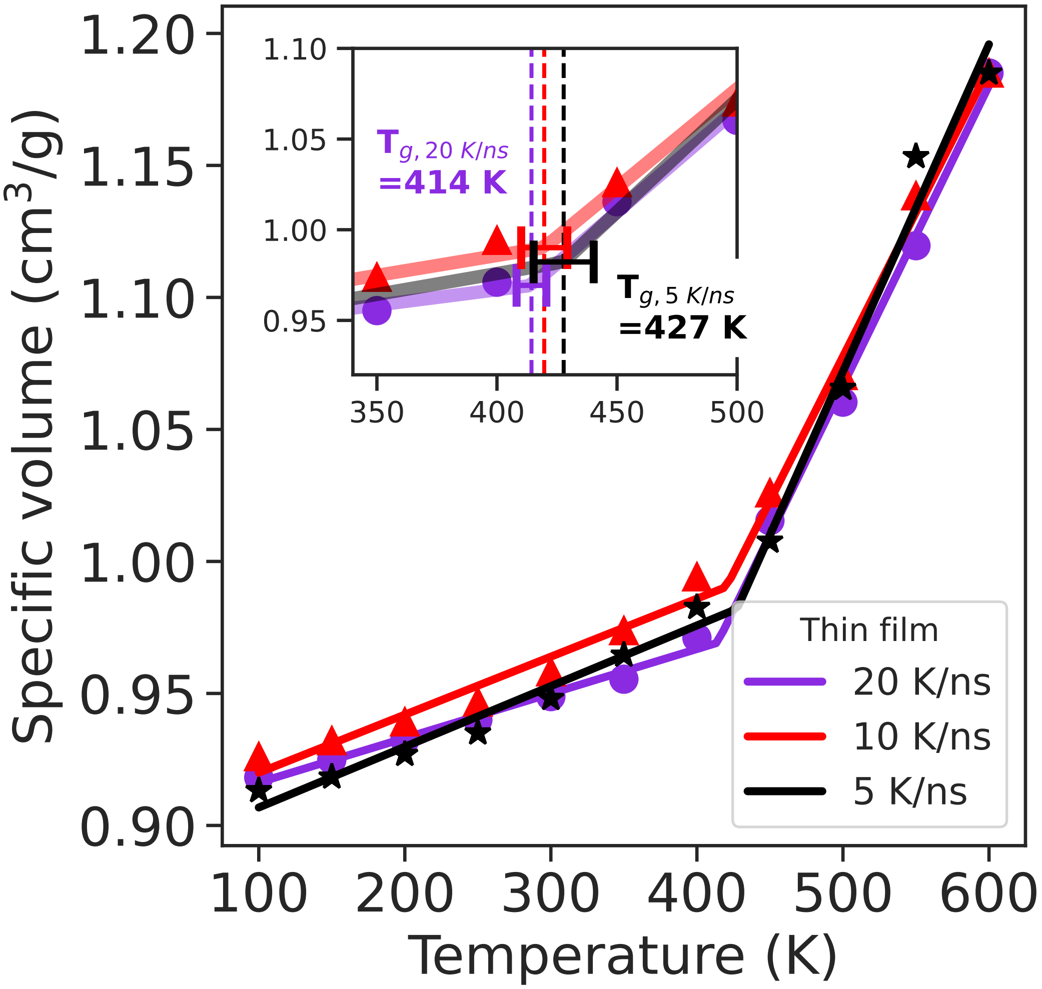


Figure S4: Effect of cooling rate on glass transition temperature of thin film with 100 P(MMA-co-EA) polymer chains. The error bars are non-linear standard error associated with the piece-wise linear fitting.

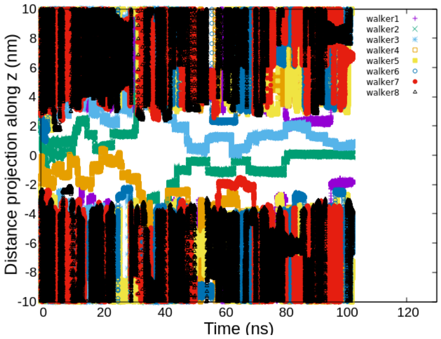


(a) acetic acid 300 K, run 1

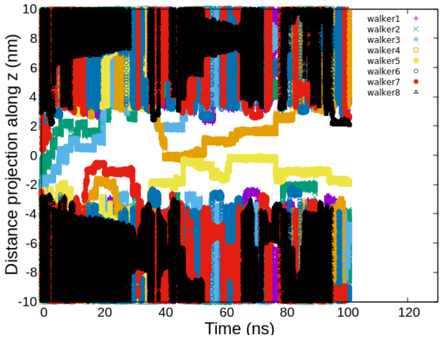


(b) acetic acid 300 K, run 2

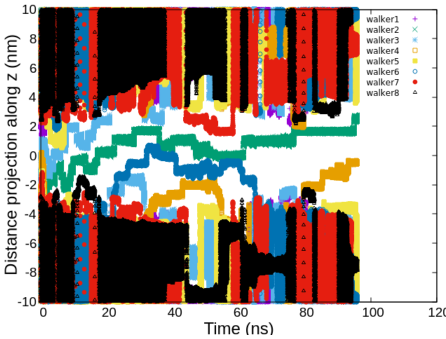


(c) formic acid 300 K, run 1

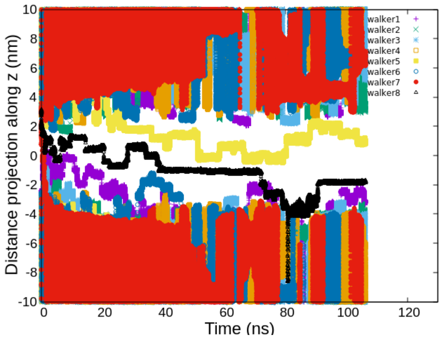


(d) formic acid 300 K, run 2

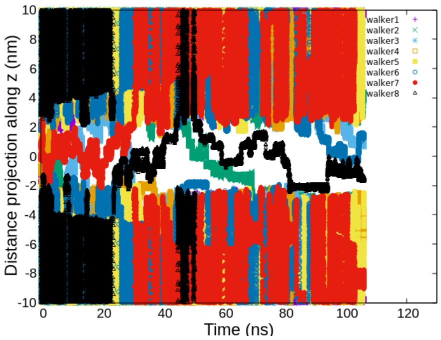


(e) formaldehyde 300 K, run 1

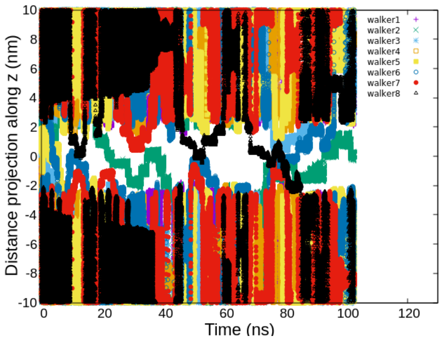


(f) formaldehyde 300 K, run 2

Figure S5: Time evolution of the collective variable, the z component of the distance between center of mass of the VOC molecule and the center of mass of the film's carbon atoms at 300 K.

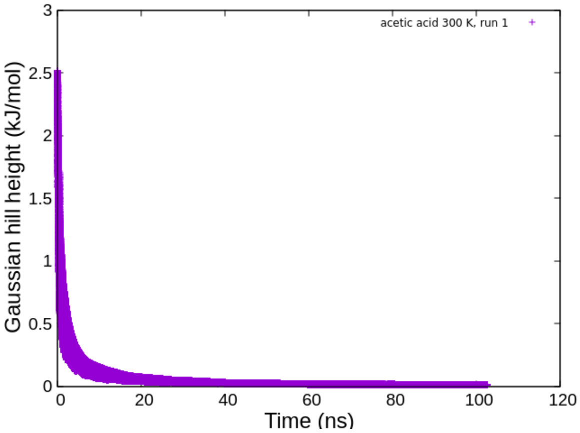


(a) acetic acid 300 K, run 1

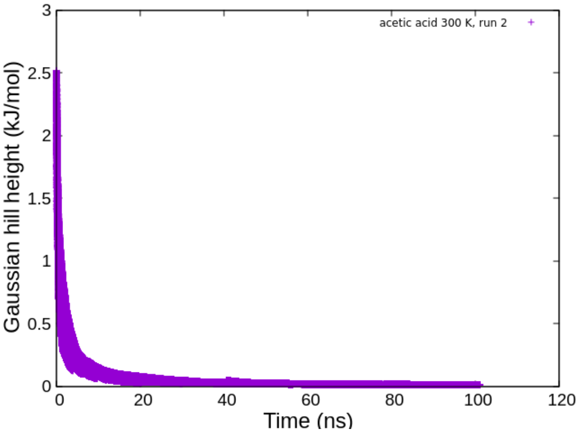


(b) acetic acid 300 K, run 2

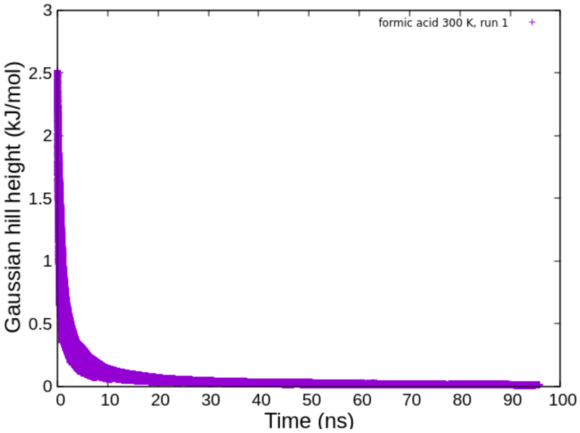


(c) formic acid 300 K, run 1

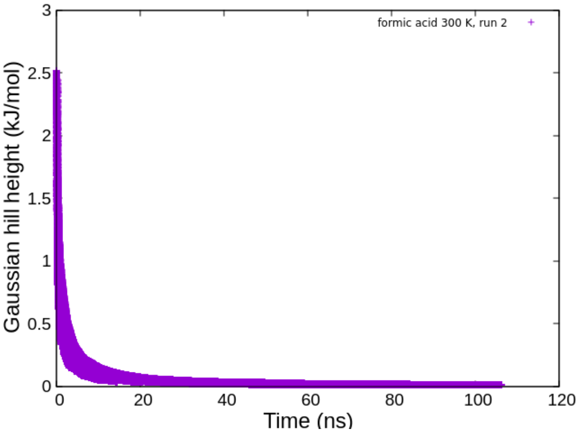


(d) formic acid 300 K, run 2

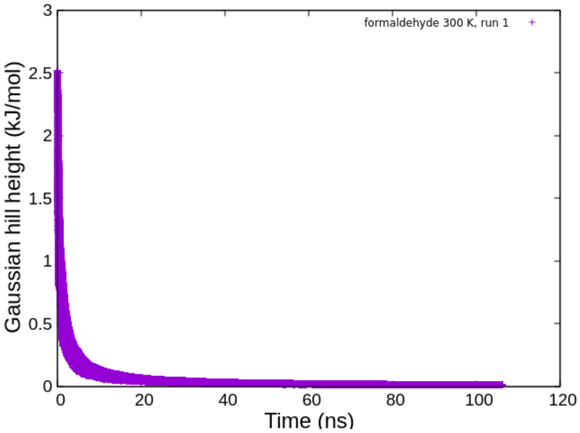


(e) formaldehyde 300 K, run 1

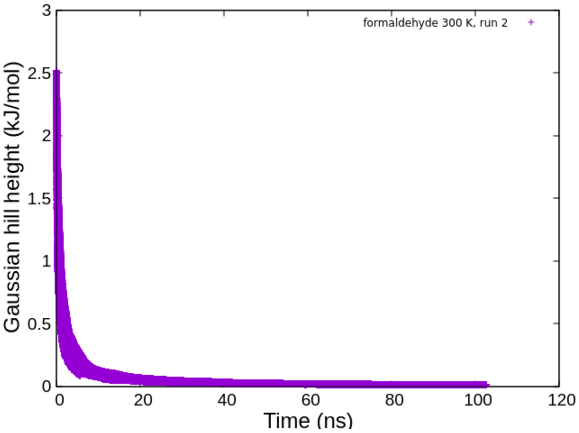


(f) formaldehyde 300 K, run 2

Figure S6: Time evolution of the Gaussian hill height at 300 K.

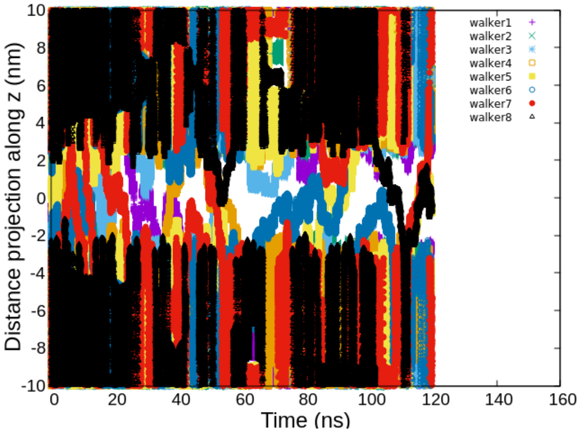


(a) acetic acid 450 K, run 1

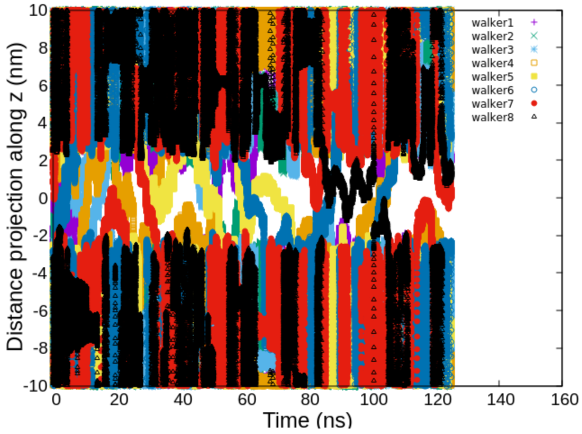


(b) acetic acid 450 K, run 2

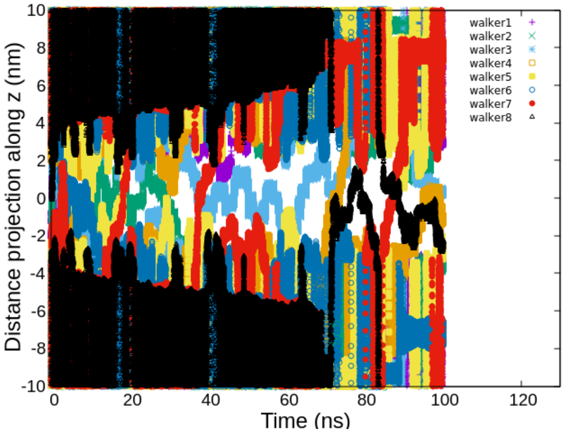


(c) formic acid 450 K, run 1

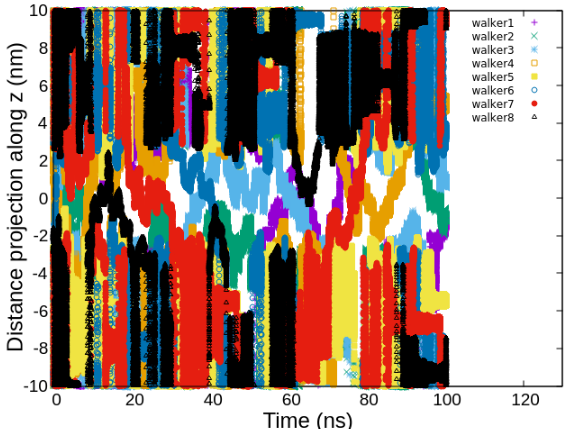


(d) formic acid 450 K, run 2

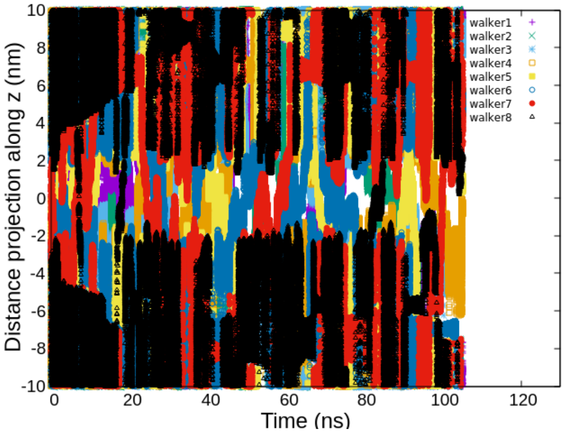


(e) formaldehyde 450 K, run 1

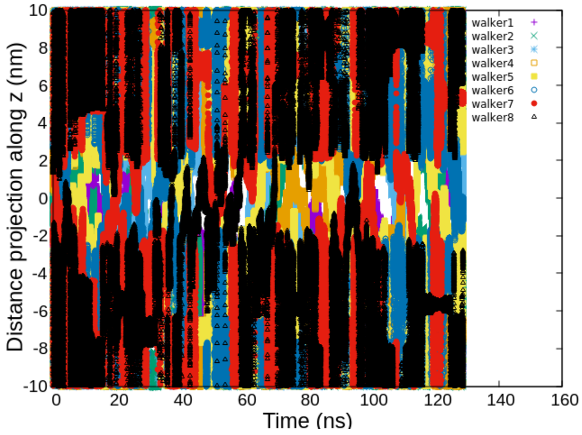


(f) formaldehyde 450 K, run 2

Figure S7: Time evolution of the collective variable, the z component of the distance between center of mass of the VOC molecule and the center of mass of the film's carbon atoms at 450 K.

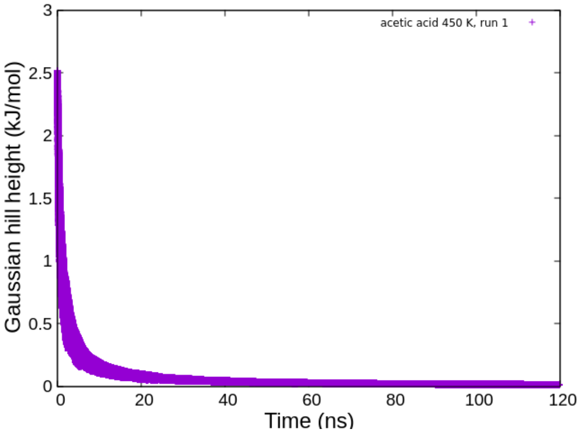


(a) acetic acid 450 K, run 1

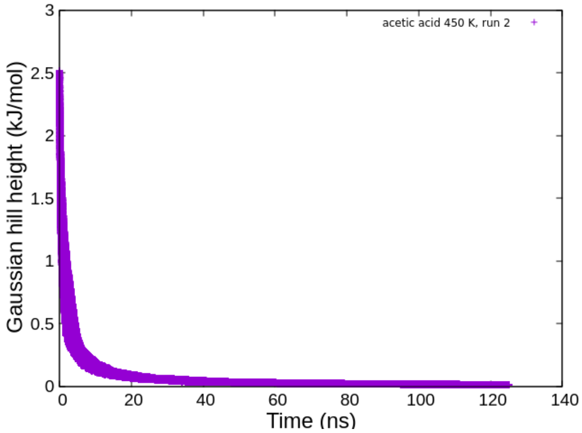


(b) acetic acid 450 K, run 2

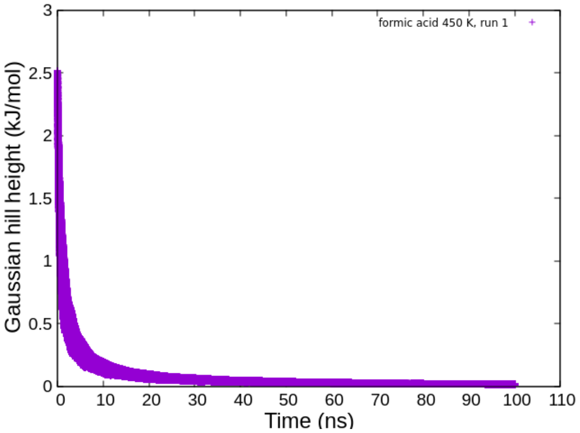


(c) formic acid 450 K, run 1

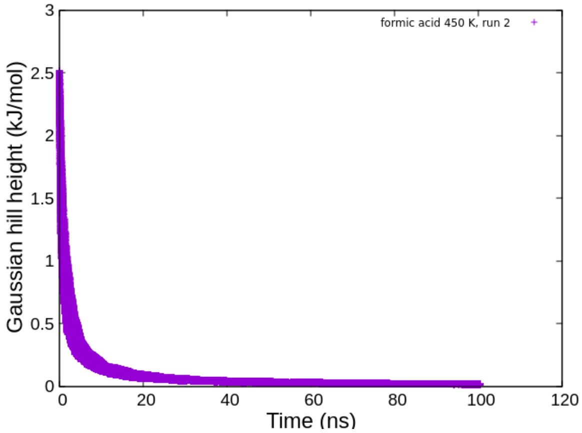


(d) formic acid 450 K, run 2

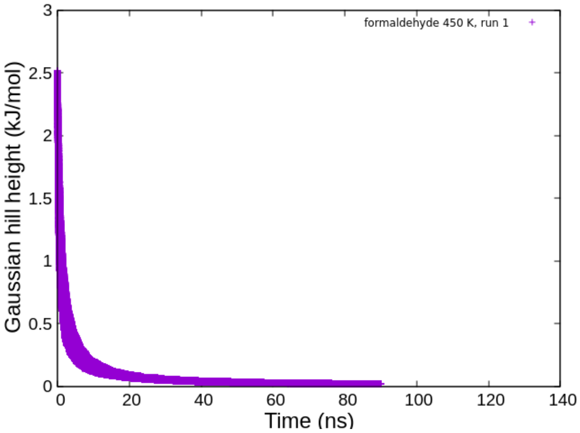


(e) formaldehyde 450 K, run 1

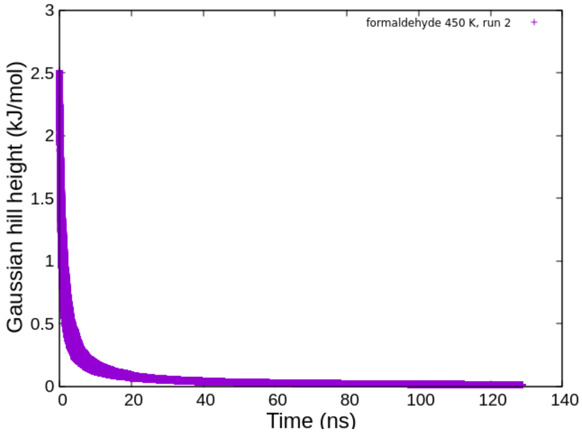


(f) formaldehyde 450 K, run 2

Figure S8: Time evolution of the Gaussian hill height at 450 K.

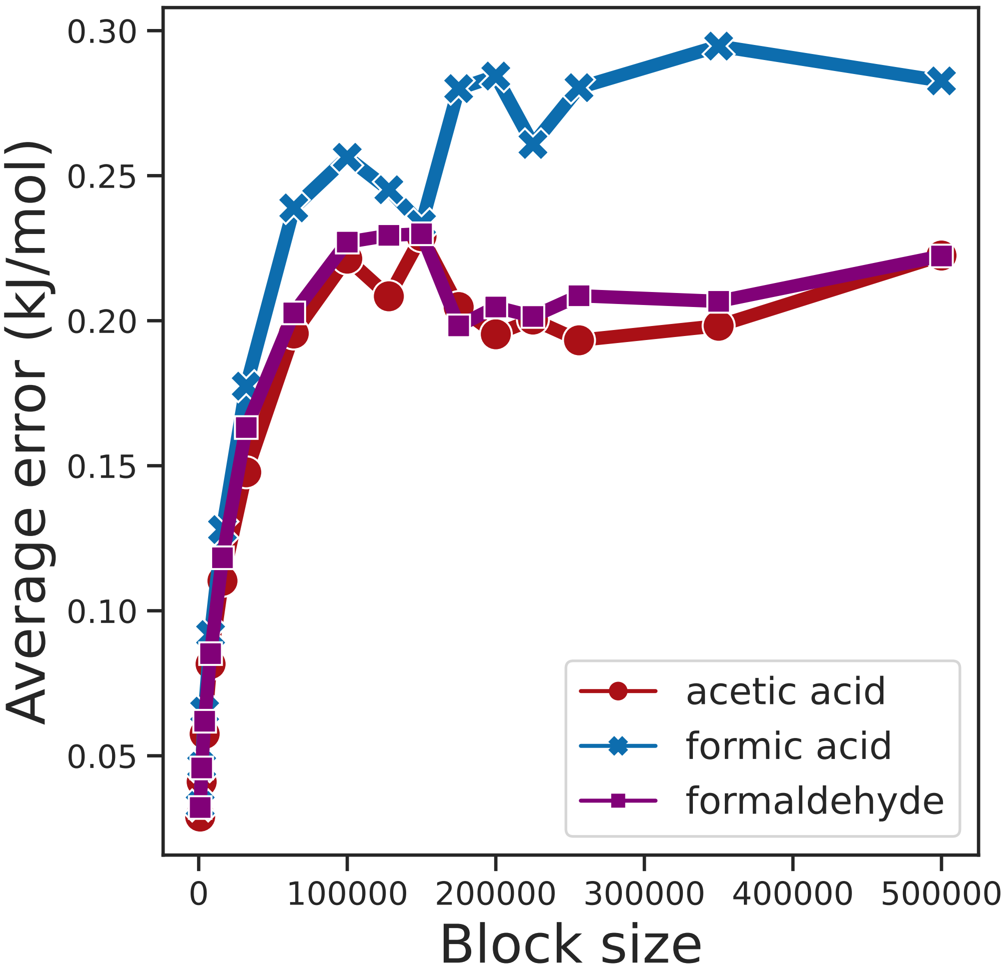


Figure S9: Dependence of average error in the free energy for simulations at 450 K on the size of the blocks used for block averaging.

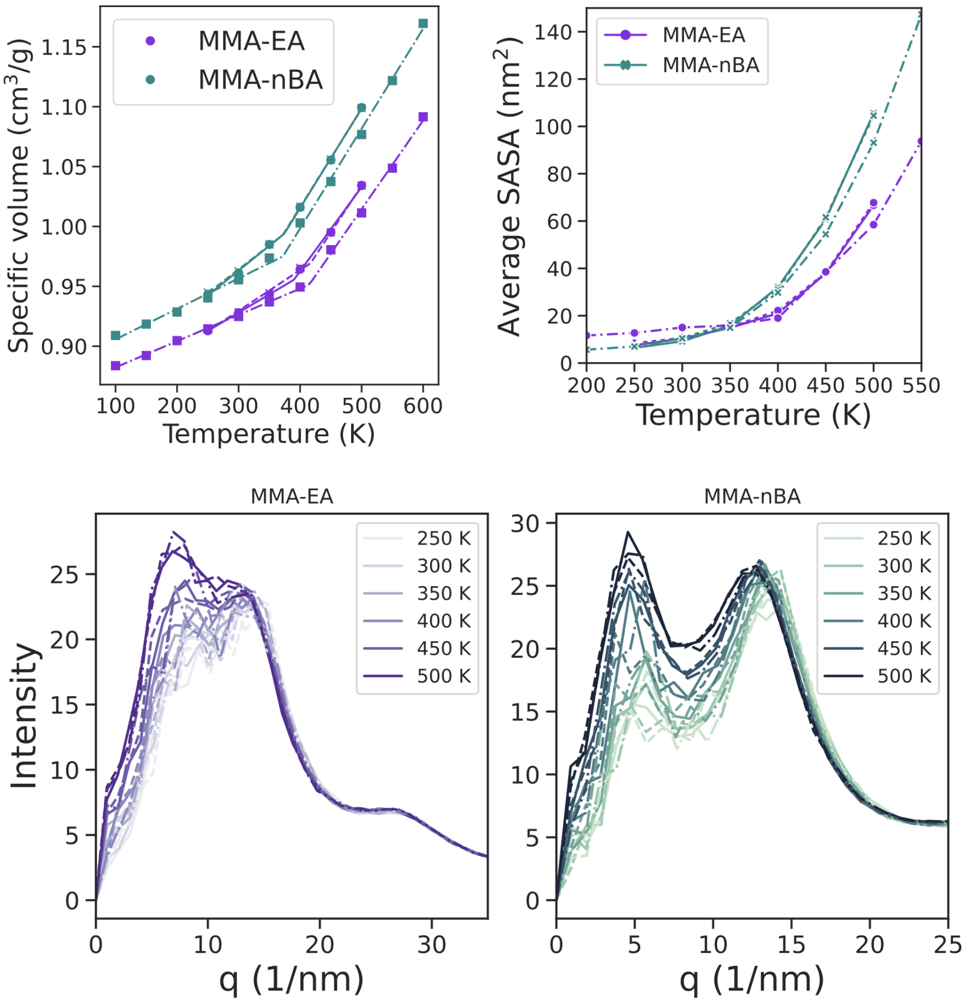


Figure S10: Comparison of structural properties for damaged copolymer chains (10mer + 5mer): glass transition behavior from specific volume vs temperature, average solvent accessible surface area (SASA) of copolymer chains and structural factors. In all plots, the solid line (———) refers to system with damaged chains (10mer + 5mer) starting from equilibrated initial configuration, dashed line (− − −) refers to system with damaged chains starting from random initial configuration, and dotdashed line (·−·−·) refers to undamaged system (15mer).

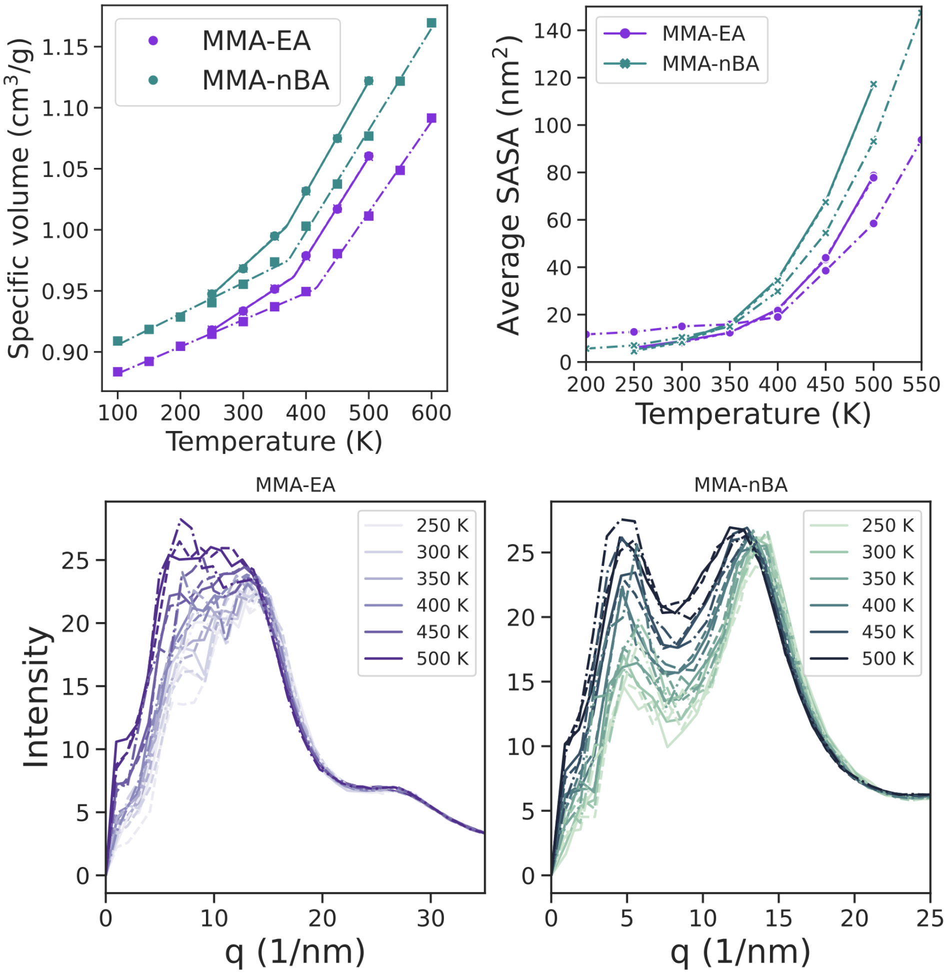


Figure S11: Comparison of structural properties for damaged copolymer chains (5mer): glass transition behavior from specific volume vs temperature, average solvent accessible surface area (SASA) of copolymer chains and structural factors. In all plots, the solid line (———) refers to system with damaged chains (5mer) starting from equilibrated initial configuration, dashed line (— — —) refers to system with damaged chains starting from random initial configuration, and dotdashed line (· — · — ·) refers to undamaged system (15mer).

# Supporting figures for properties of acrylic thin films

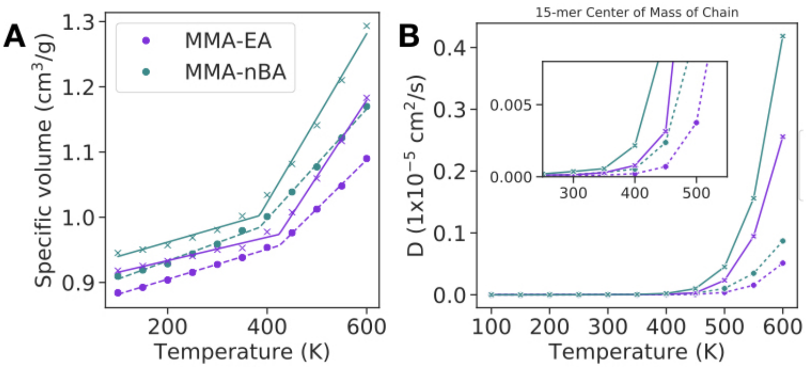


Figure S12: (A) Glass transition temperature and (B) self-diffusion coefficients calculated for P(MMA-co-EA) and P(MMA-co-$n$BA). Only data from the last 10 ns of 50 ns simulation is used for the calculation of self-diffusion coefficients. Solid lines indicate the film phase while dashed lines indicate bulk phase.

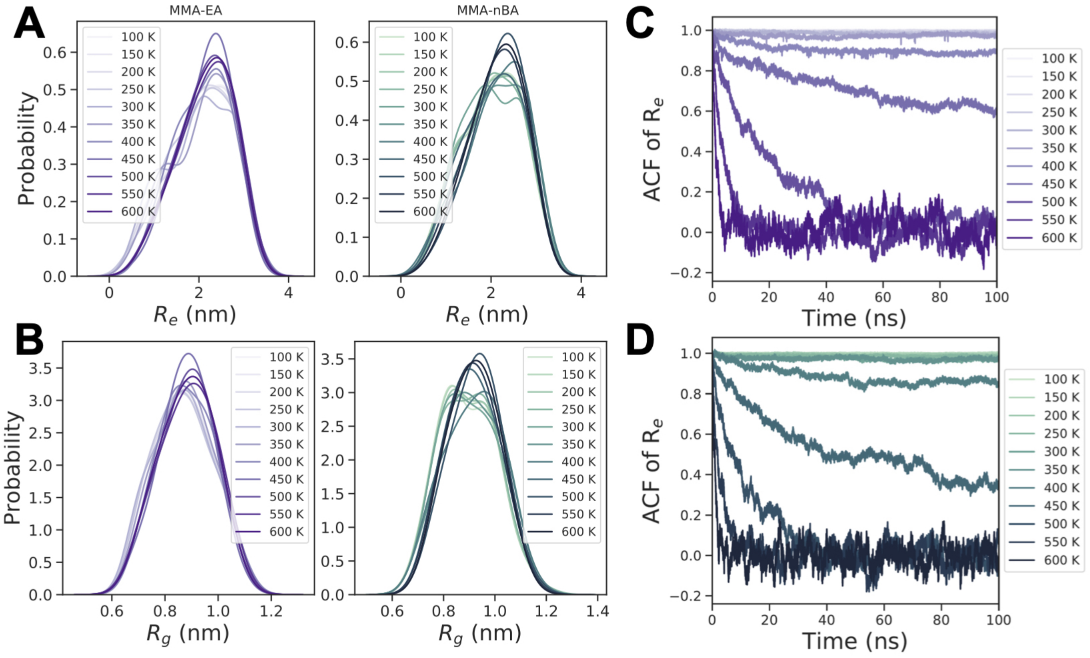


Figure S13: Probability distribution of (**A**) end-to-end distance, $R_e$, and (**B**) radius of gyration, $R_g$, and (**C,D**) end-to-end vector autocorrelation function for P(MMA-co-EA) and P(MMA-co-nBA) in film simulations at different temperatures.

# Supporting figures for pollutant absorption from the environment

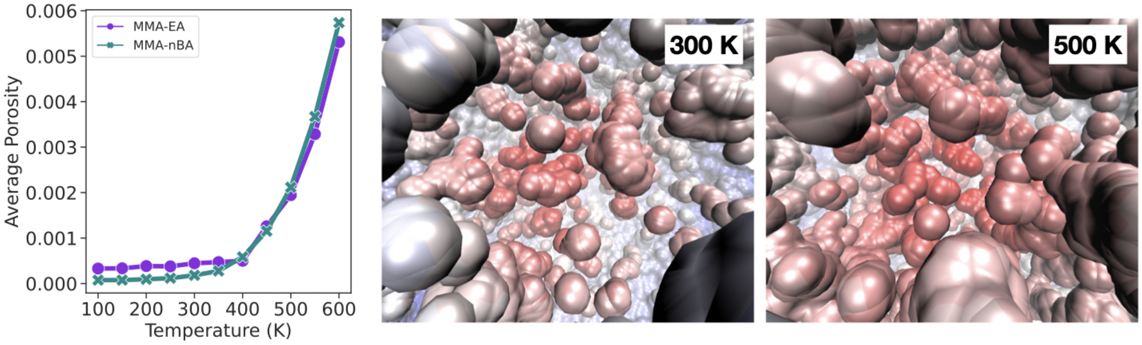


Figure S14: The average porosity of copolymers P(MMA-co-EA) and P(MMA-co-nBA) as a function of temperature and representative snapshots at 300 K and 500 K showing the surface of the pores in the acrylics. Porosity is calculated as the ratio of free volume in the polymer to the total volume using a probe radius of 0.14 nm, roughly corresponding to a water molecule.

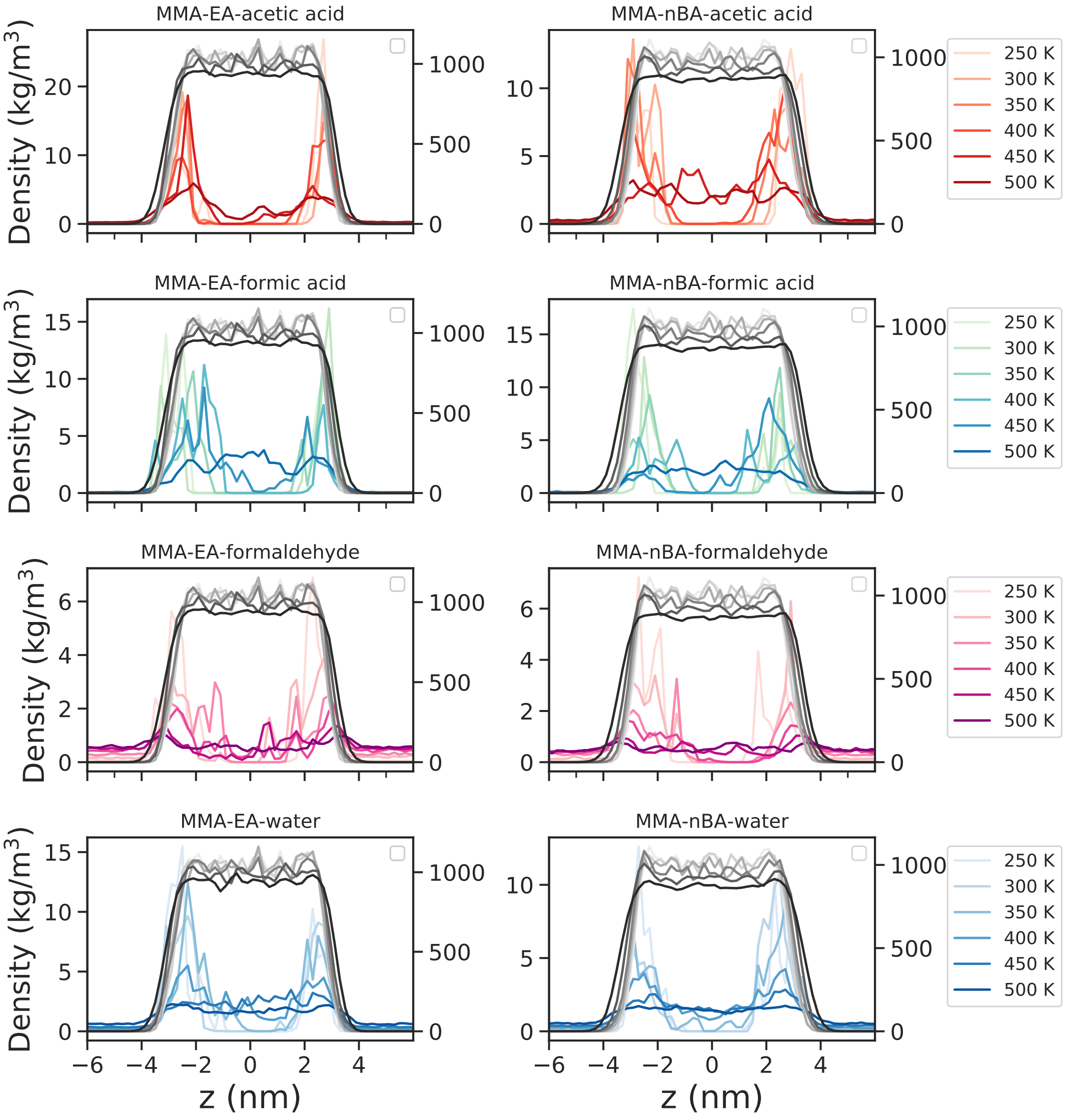


Figure S15: Average density of the VOCs, water and copolymers along the z-axis (thickness) of the film calculated for P(MMA-co-EA) and P(MMA-co-$n$BA) at different temperatures. Density of VOCs and water is shown on the left side, while the density of the copolymers (grey) is shown on the right side of the y-axis.

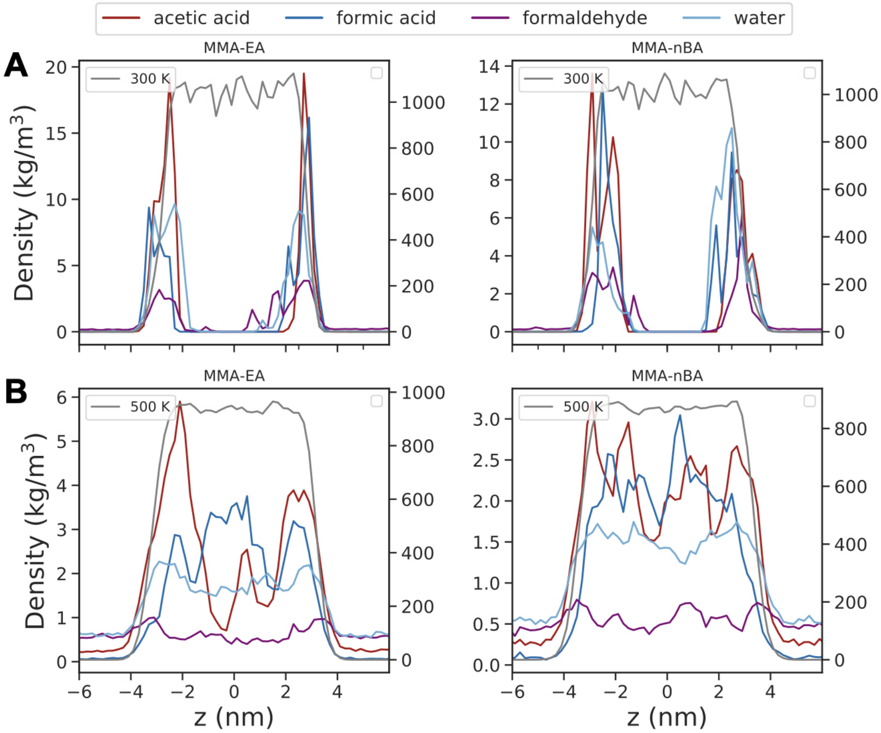


Figure S16: Comparing the distribution of VOCs and water at (**A**) 300 and (**B**) 500 K. Average density of VOCS, water and copolymers along the z-axis (thickness) of the film calculated for P(MMA-co-EA) and P(MMA-co-$n$BA) separately for 300 and 500 K. Density of VOCs is shown on the left side, while the density of the copolymers (grey) is shown on the right side of the y-axis.

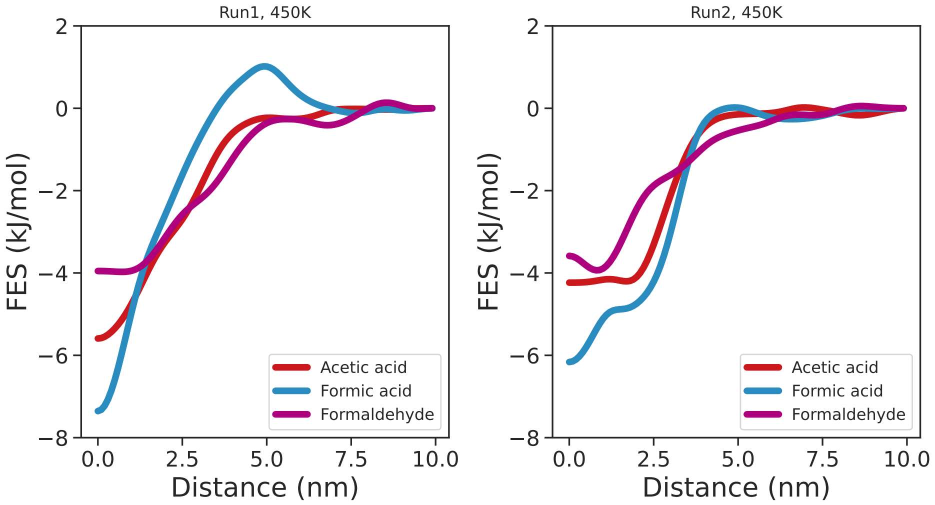


Figure S17: The free energy surface (FES) as a function of distance of pollutants from the center of the P(MMA-co-EA) polymer film at 450 K from two independent simulations.

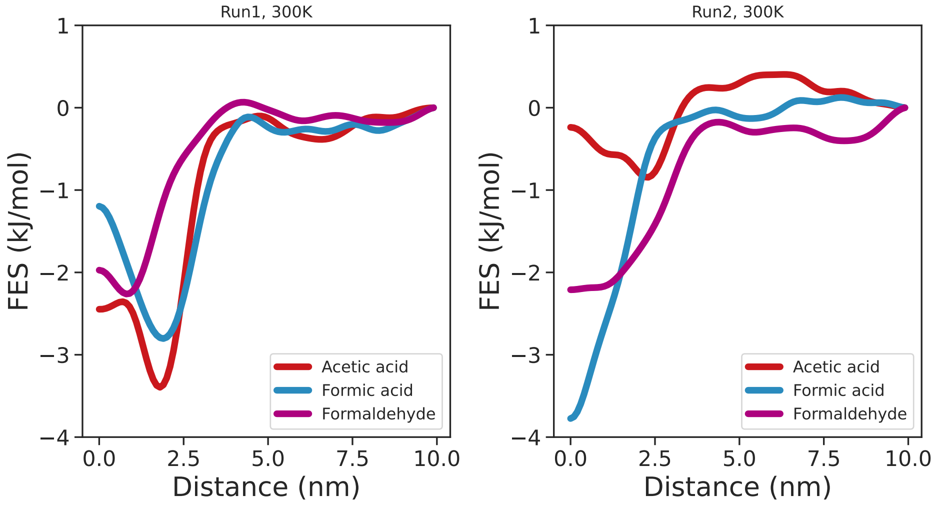


Figure S18: The free energy surface (FES) as a function of distance of pollutants from the center of the P(MMA-co-EA) polymer film at 300 K from two independent simulations.

# Supporting figures for degradation in acrylics

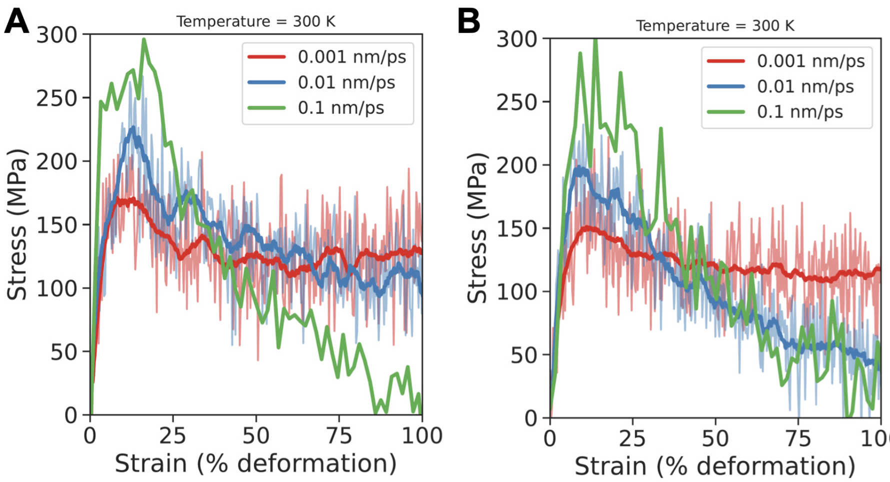


Figure S19: Effect of straining rate at constant temperature (300 K) on stress versus strain curves for (A) P(MMA-co-EA) and (B) P(MMA-co-nBA).

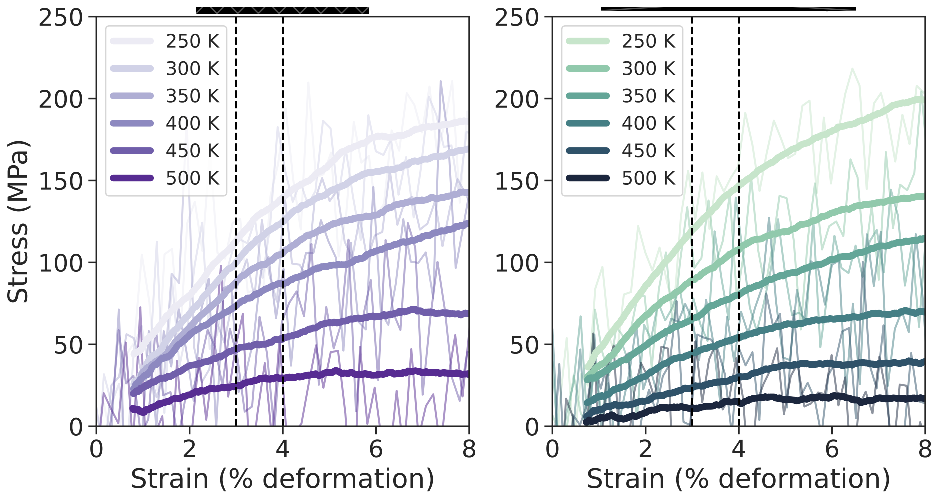


Figure S20: Effect of temperature at constant strain rate (0.001 nm/ps) on stress versus strain curves for (left) P(MMA-co-EA) and (right) P(MMA-co-nBA). Only the elastic region of the stress-strain curves are plotted to show the linear dependence up to 4% strain.

## Supporting figures for degradation by absorption of environmental pollutants

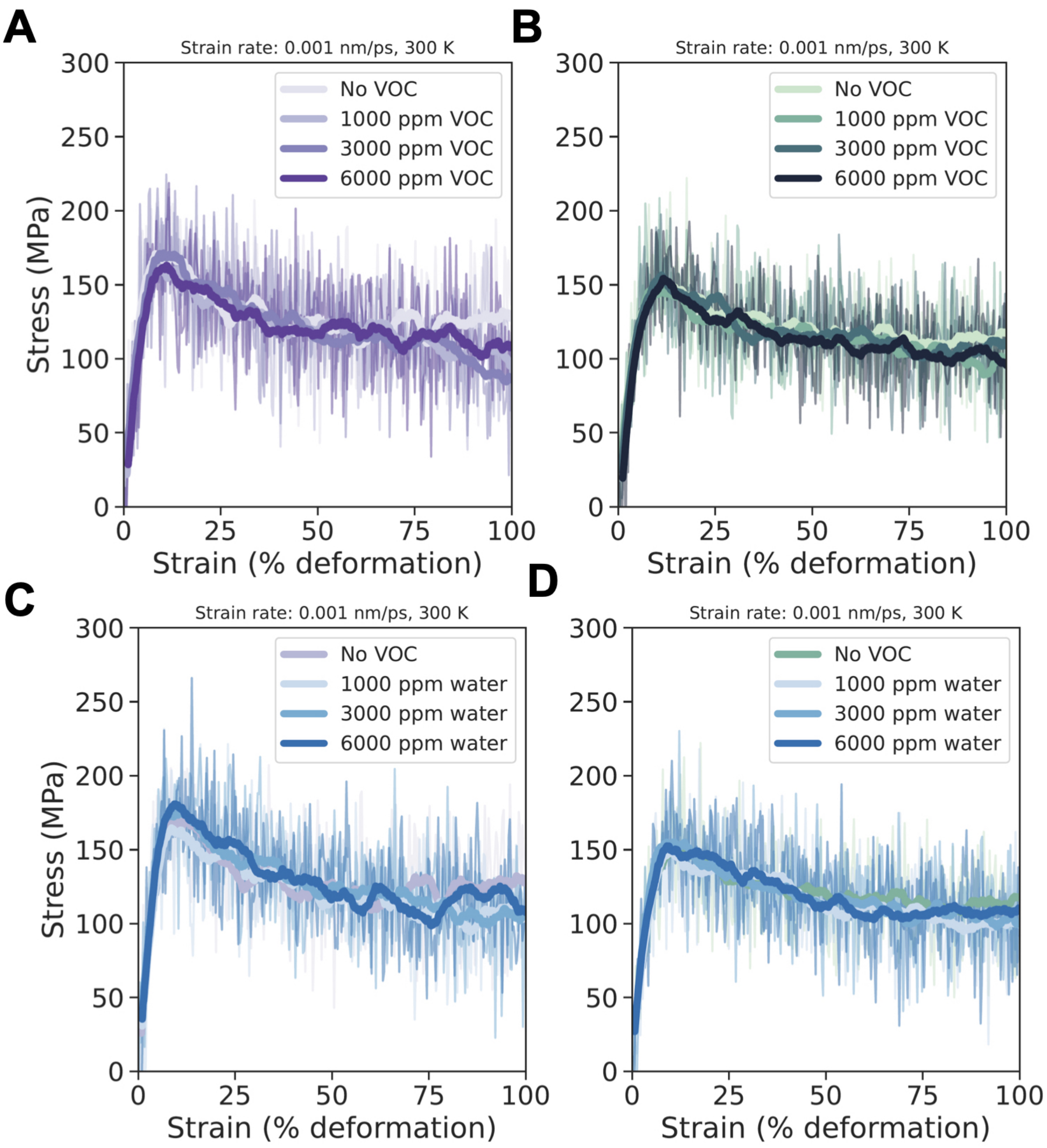


Figure S21: Stress versus strain curves for P(MMA-co-EA) (left) and P(MMA-co-nBA) (right) as a function of (A-B) VOC concentration and (C-D) water concentration (relative humidity) at 300 K with strain rate of 0.001 nm/ps.

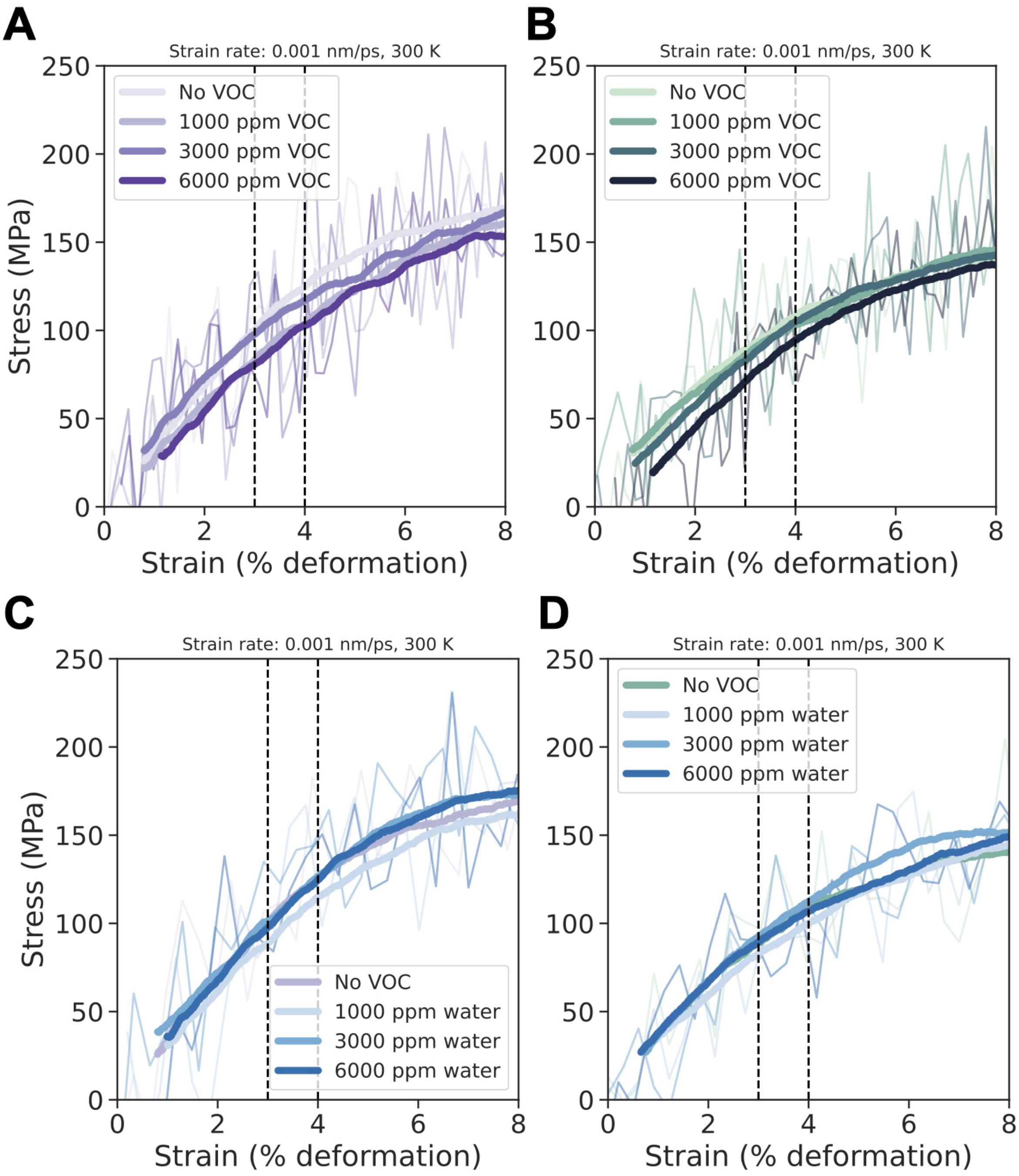


Figure S22: Elastic region of stress versus strain curves for P(MMA-co-EA) (left) and P(MMA-co-nBA) (right) as a function of (A-B) VOC concentration and (C-D) water concentration (relative humidity) at 300 K with strain rate of 0.001 nm/ps.

## Supporting figures for degradation by bond scission

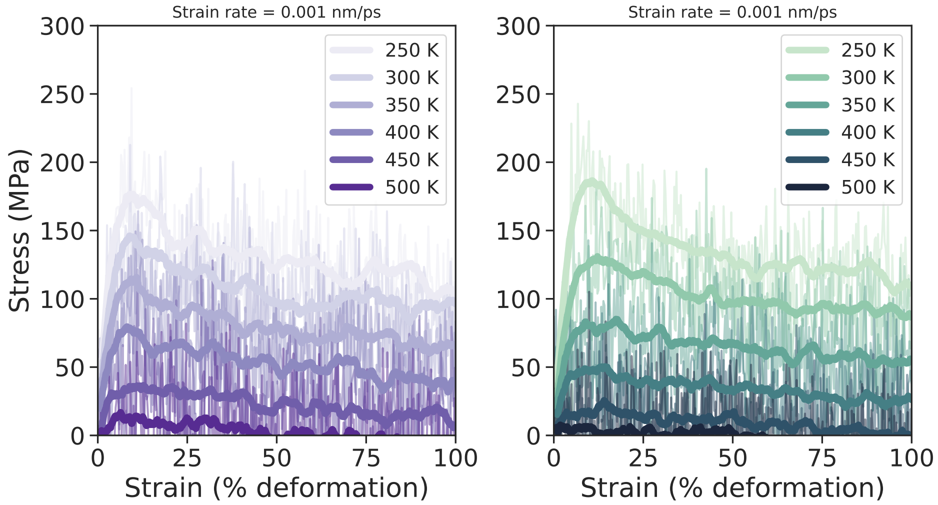


(a) 10mer+5mer

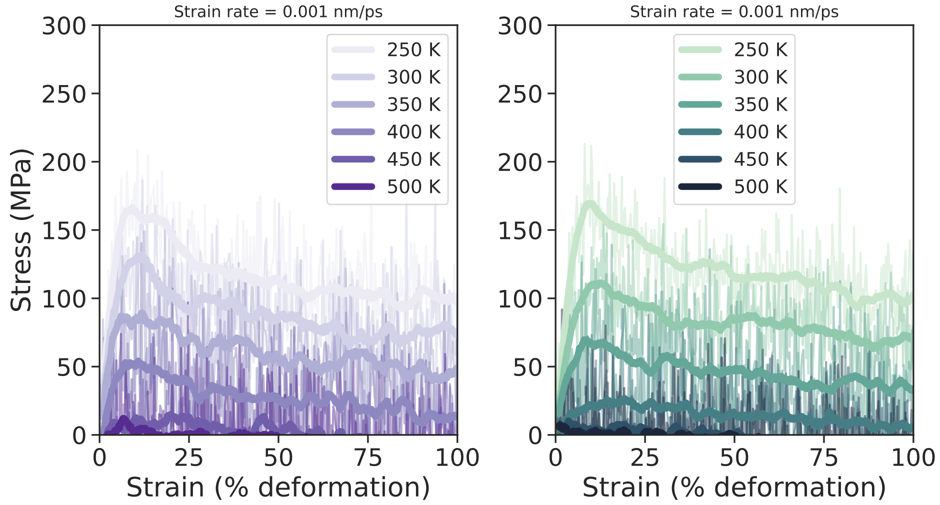


(b) 5mer only

Figure S23: Stress versus strain curves for P(MMA-co-EA) (left) and P(MMA-co-nBA) (right) as a function of temperature after bond scission damage with strain rate of 0.001 nm/ps.

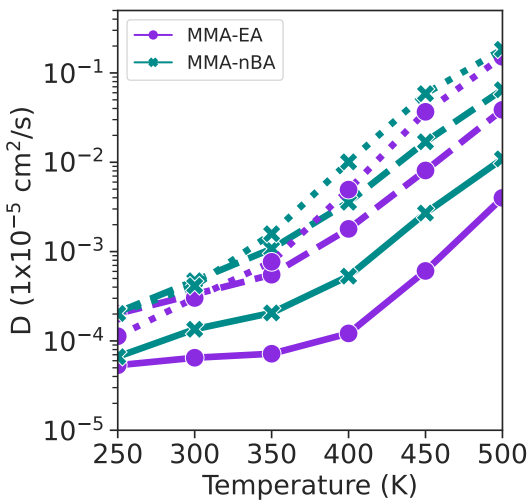


Figure S24: The self-diffusion coefficients of polymer chains for P(MMA-co-EA) and P(MMA-co-nBA) as a function of temperature for 10mer + 5mer (– – –) and 5mer (· · · · ·) polymer chains after bond scission compared to 15mer polymer chains (———).

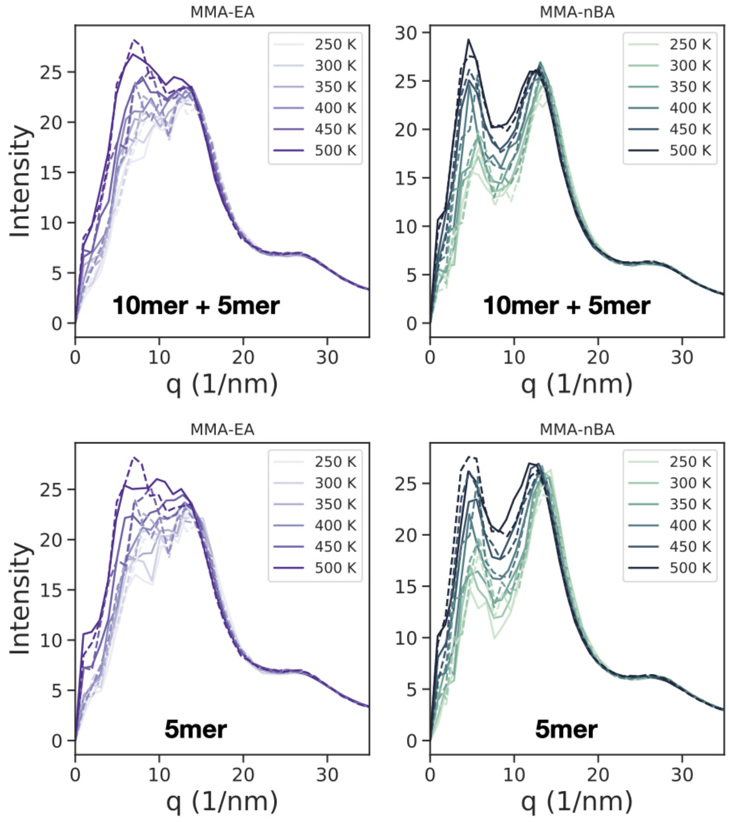


Figure S25: Small angle X-ray scattering structure factor P(MMA-co-EA) and P(MMA-co-$n$BA) at different temperatures. The top panel shows the effect of bond scission after the first cut (10mer + 5mer) and the bottom panel shows the effect after the second cut (5mer only). Solid lines refer to data from damaged chains, while dashed lines refer to simulation with undamaged 15mer copolymer chains.

# Supporting figures for degradation by intermolecular cross-linking

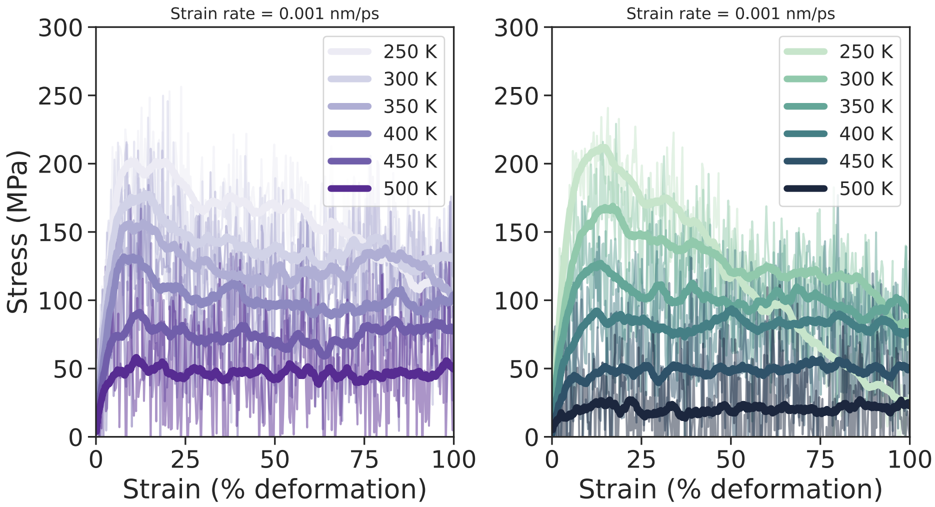


(a) 5% cross-linking

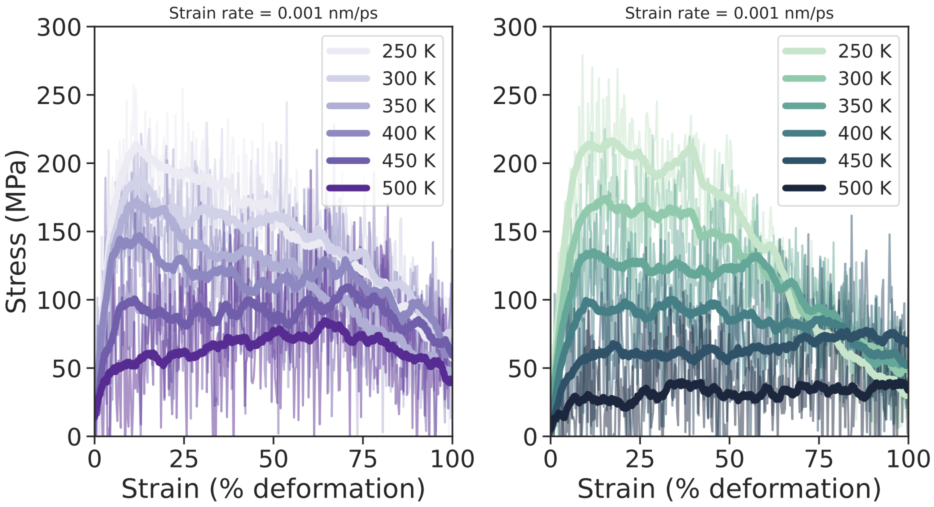


(b) 10% cross-linking

Figure S26: Stress versus strain curves for P(MMA-co-EA) (left) and P(MMA-co-nBA) (right) as a function of temperature after 5% and 10% cross-linking with strain rate of 0.001 nm/ps.

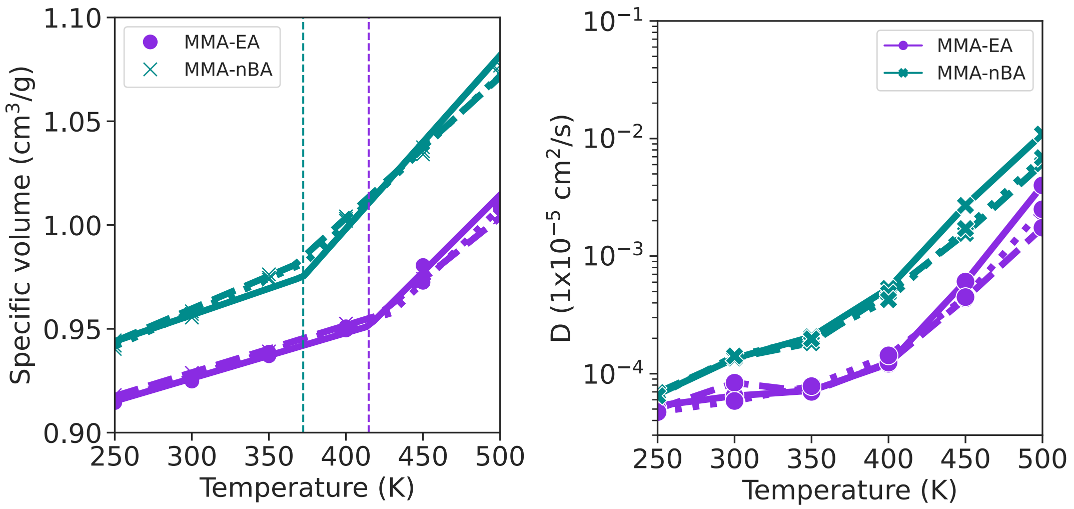


(a) (b)

Figure S27: The glass transition behavior from specific volume and self-diffusion coefficients of polymer chains for P(MMA-co-EA) and P(MMA-co-nBA) as a function of temperature with 5% (· · · · ·) and 10% (− − −) cross-linking compared to polymer chains with no cross-linking (———).

# Supporting figures for pollutant diffusion in degraded polymers

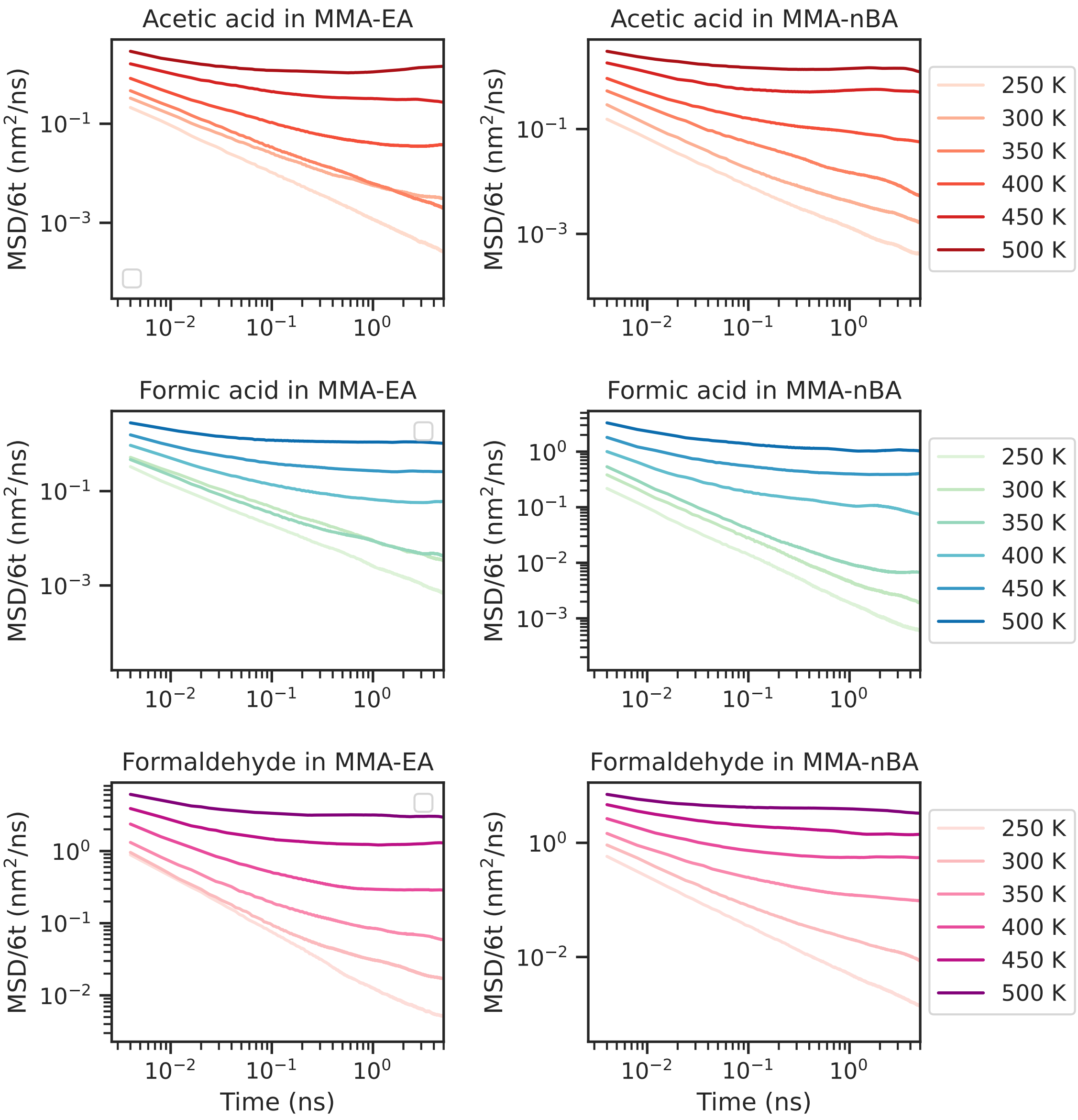


Figure S28: The mean square displacement (MSD) of center of mass of 1000 ppm acetic acid, formic acid and formaldehyde divided by 6×time in damaged (5mer) P(MMA-co-EA) and P(MMA-co-$n$BA) over 10 ns of trajectory. Diffusion coefficients are calculated at long time, where MSD/6t reaches a constant value. Below $T_g$, the pollutants do not diffuse freely due to glassy behavior of the polymer

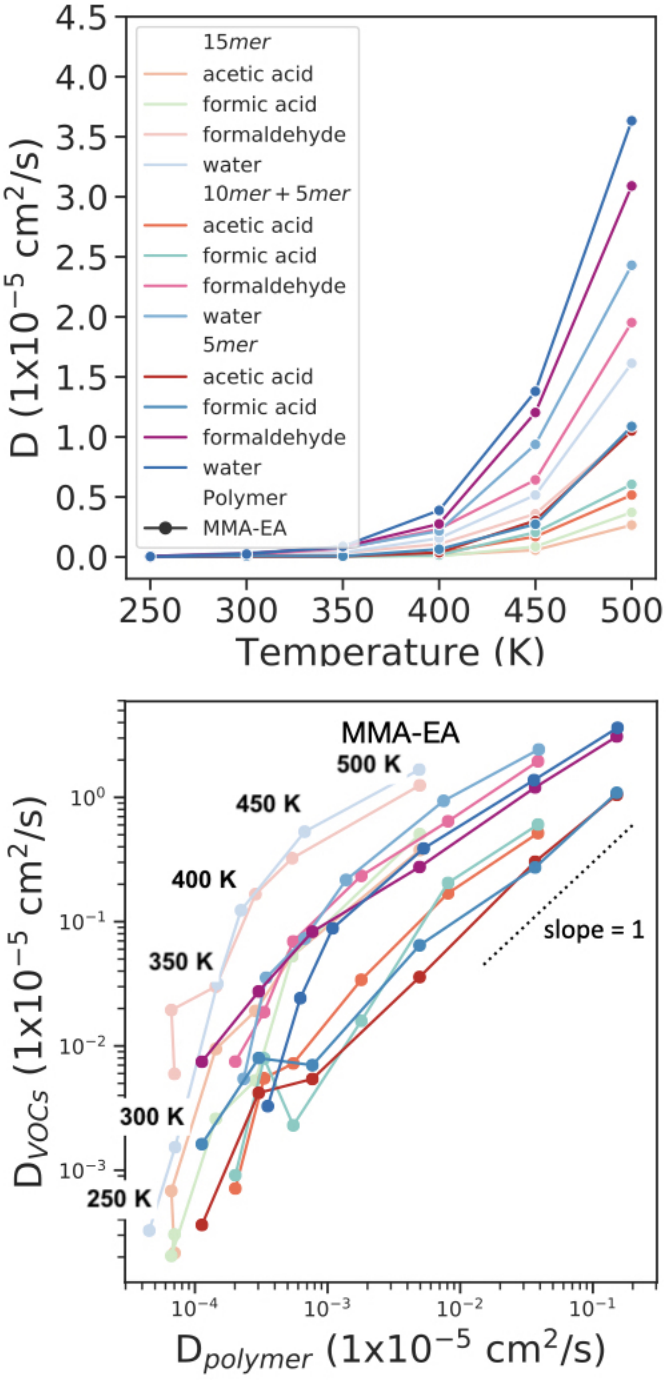


Figure S29: The self-diffusion coefficients of acetic acid, formic acid, formaldehyde and water in P(MMA-co-EA) (left) and P(MMA-co-*n*BA) (right) as a function of temperature. Relationship between the diffusion coefficients of VOCs/water and polymer chains for P(MMA-co-EA) (right) and P(MMA-co-nBA) (left) are shown on the bottom panel. Diffusion coefficients increase with temperature. Temperature labels are shown for water diffusivities.

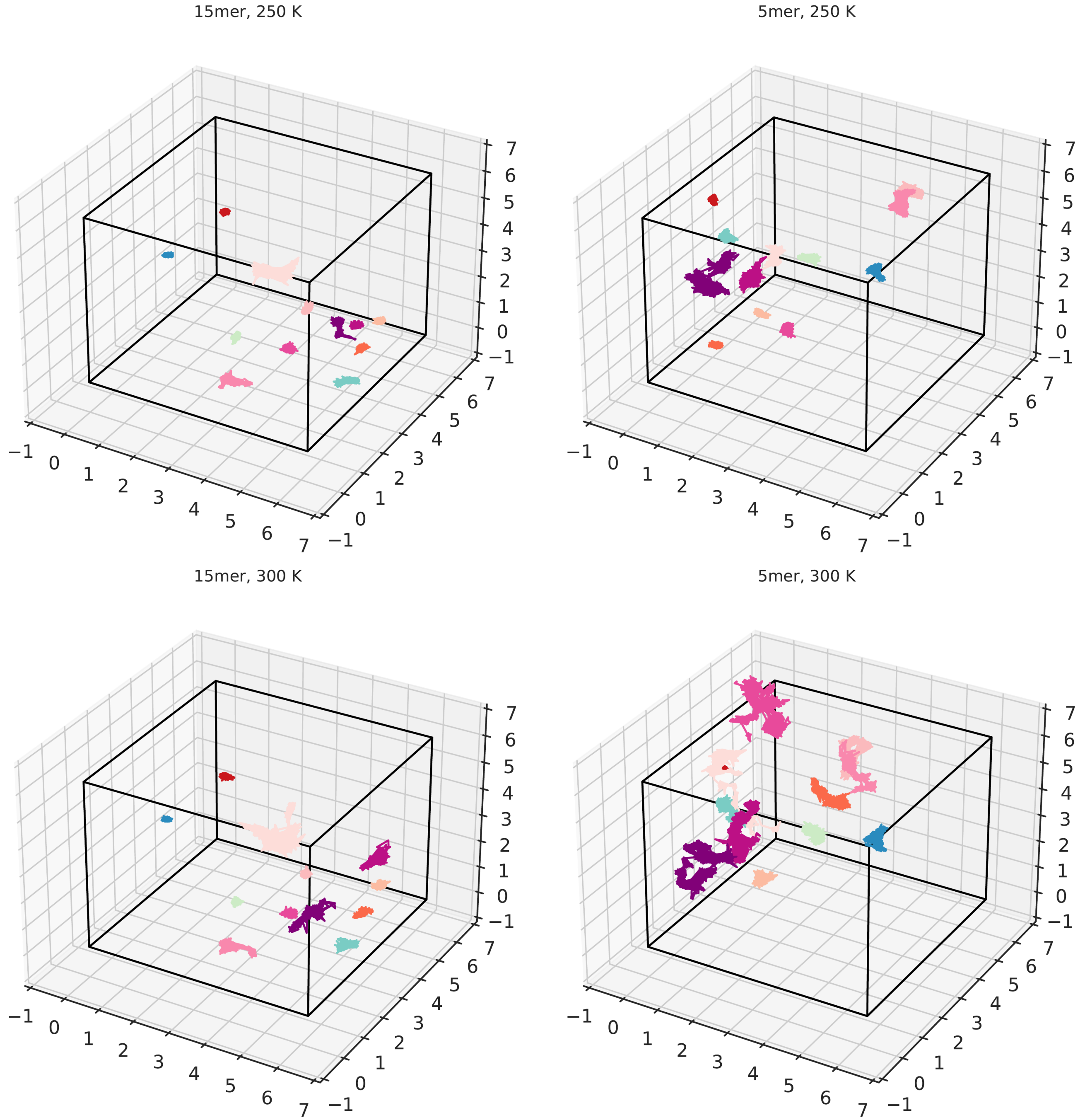


Figure S30: The center of mass trajectories of acetic acid, formic acid and formaldehyde at 250 K and 300 K in undamaged (15mer) and damaged (5mer) P(MMA-co-EA).

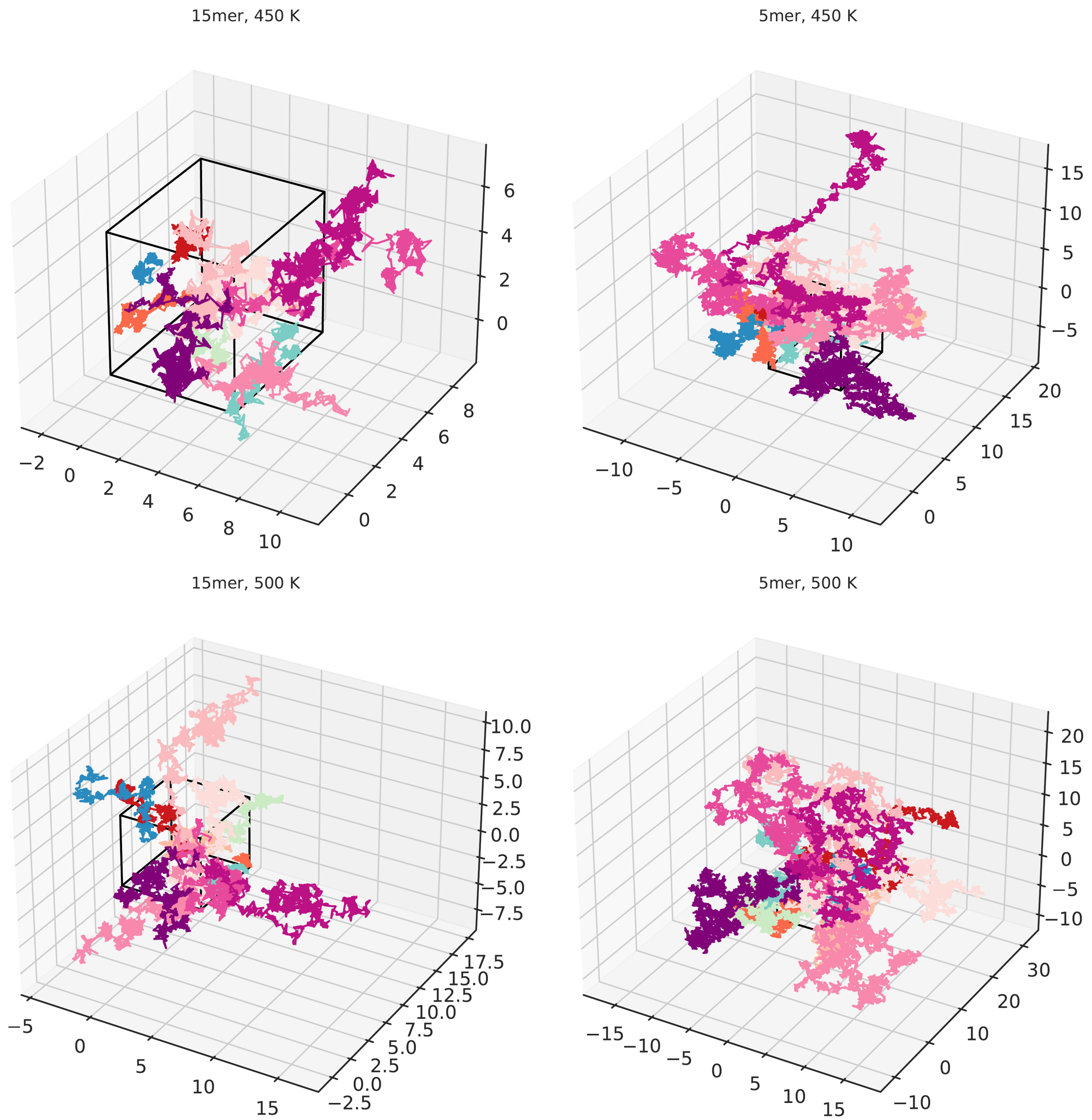


Figure S31: The center of mass trajectories of acetic acid, formic acid and formaldehyde at 450 K and 500 K in undamaged (15mer) and damaged (5mer) P(MMA-co-EA).

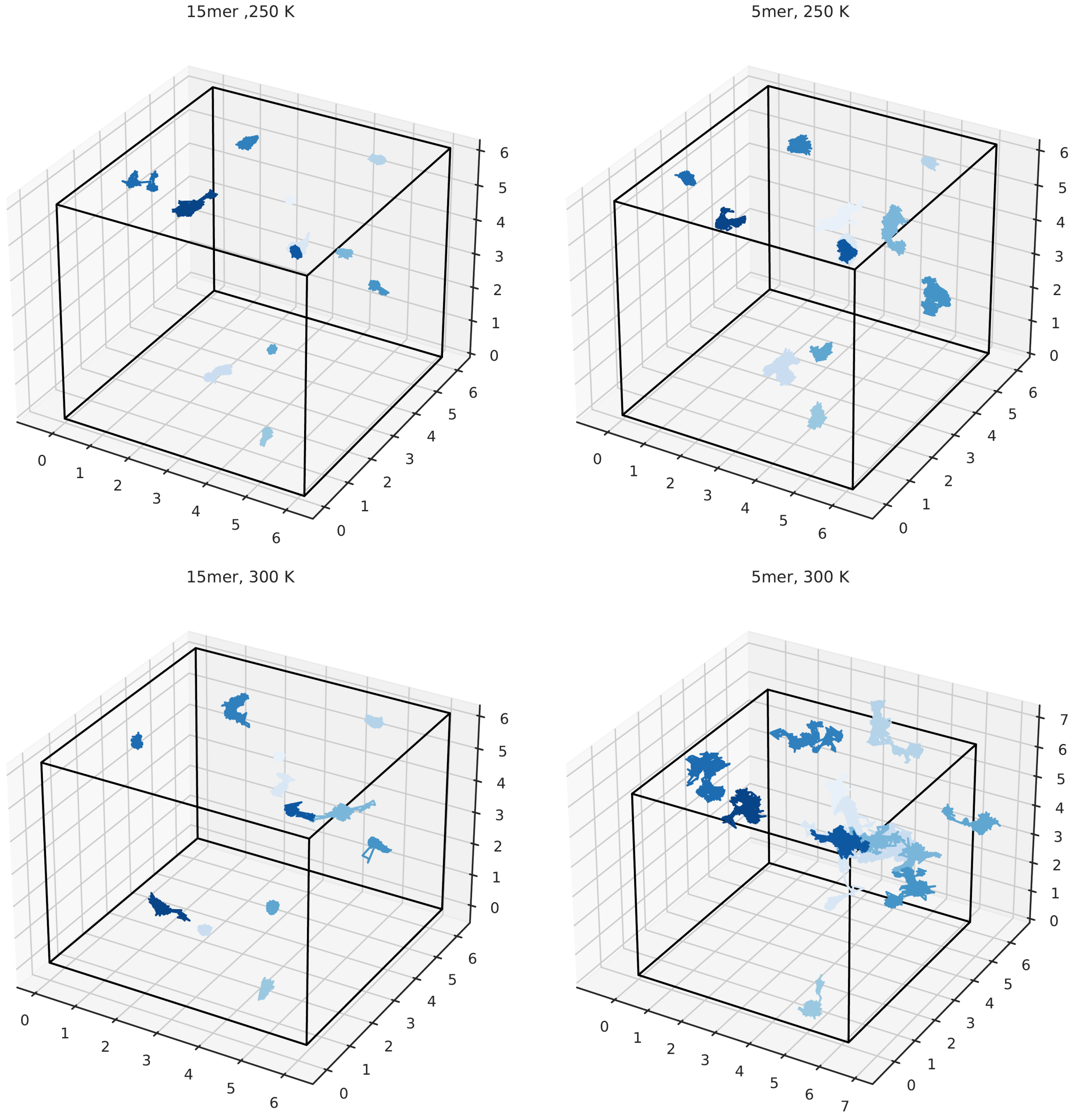


Figure S32: The center of mass trajectories of water at 250 K and 300 K in undamaged (15mer) and damaged (5mer) P(MMA-co-EA).

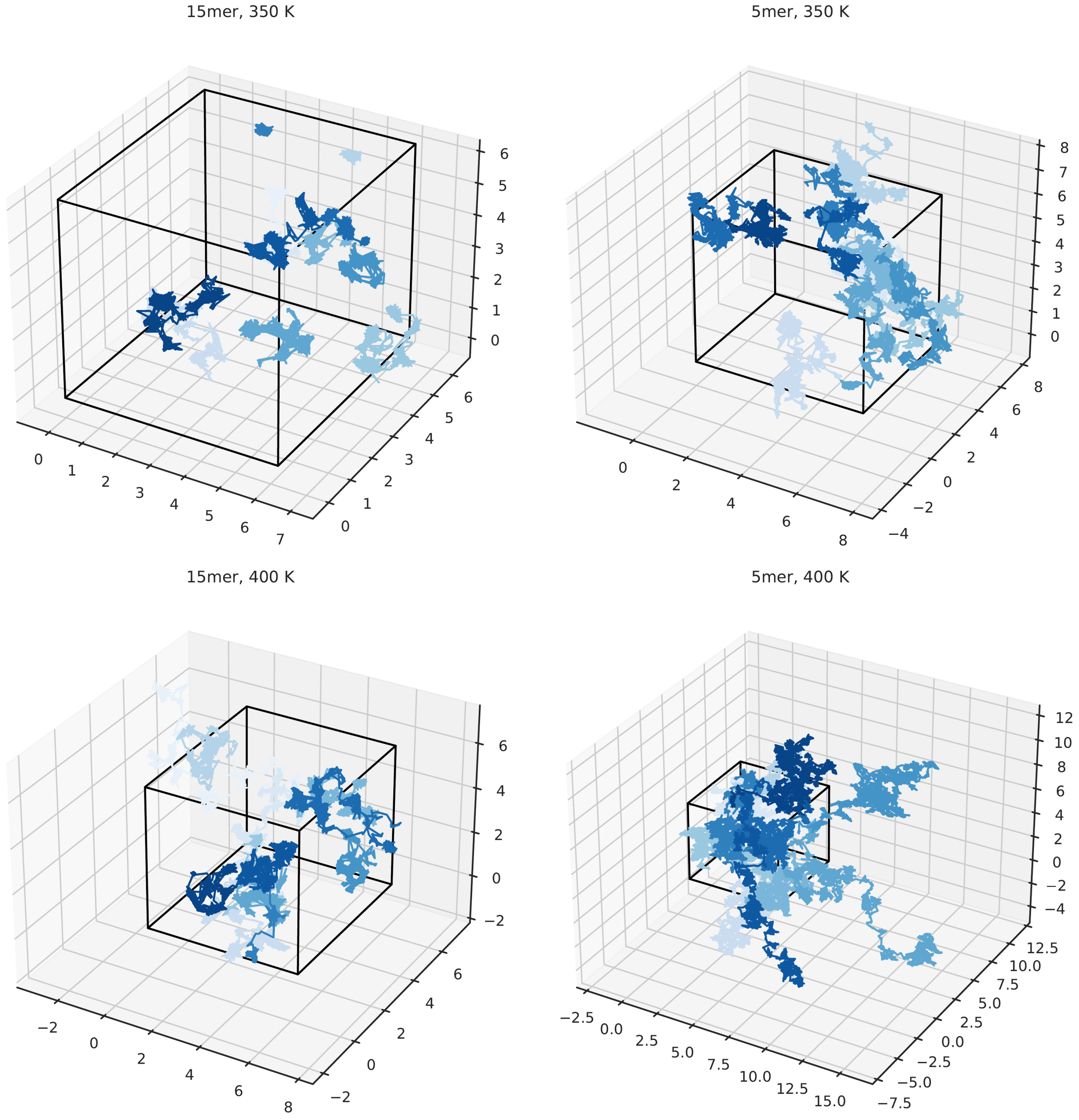


Figure S33: The center of mass trajectories of water at 350 K and 400 K in undamaged (15mer) and damaged (5mer) P(MMA-co-EA).

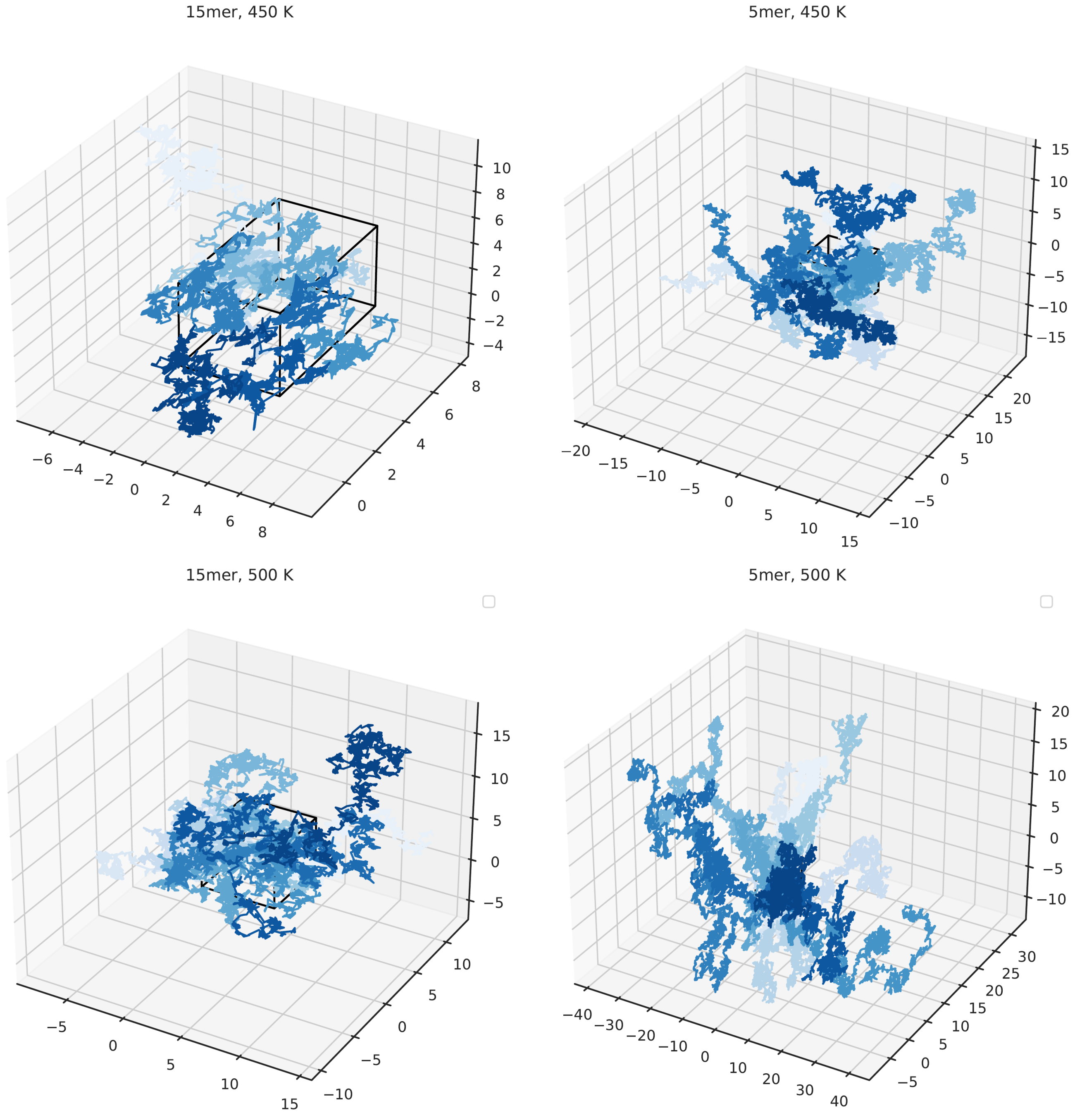


Figure S34: The center of mass trajectories of water at 450 K and 500 K in undamaged (15mer) and damaged (5mer) P(MMA-co-EA).
